# Non‐Planar Structures of Sterically Overcrowded Trialkylamines

**DOI:** 10.1002/chem.202003933

**Published:** 2020-12-14

**Authors:** Klaus Banert, Manuel Heck, Andreas Ihle, Tharallah Shoker, Michael Wörle, A. Daniel Boese

**Affiliations:** ^1^ Organic Chemistry Chemnitz University of Technology Strasse der Nationen 62 09111 Chemnitz Germany; ^2^ Department of Chemistry and Applied Biosciences ETH Zürich Vladimir-Prelog-Weg 1 8093 Zürich Switzerland; ^3^ Institute of Chemistry, Physical and Theoretical Chemistry University of Graz Heinrichstrasse 28/IV 8010 Graz Austria

**Keywords:** amines, molecular structures, quantum chemistry, steric hindrance, X-ray diffraction

## Abstract

Several amines with three bulky alkyl groups at the nitrogen atom, which exceed the steric crowding of triisopropylamine significantly, were synthesized, mainly by treating *N*‐chlorodialkylamines with Grignard reagents. In six cases, namely *tert*‐butyldiisopropylamine, 1‐adamantyl‐*tert*‐butylisopropylamine, di‐1‐adamantylamines with an additional *N*‐cyclohexyl or *N‐exo*‐2‐norbonyl substituent, as well as 2,2,6,6‐tetramethylpiperidine derivatives with *N*‐cyclohexyl or *N*‐neopentyl groups, appropriate single crystals were generated that enabled X‐ray diffraction studies and analysis of the molecular structures. The four noncyclic amines adopt triskele‐like conformations, and the sum of the three C−N−C angles is always in the range of 351.1° to 352.4°. Consequently, these amines proved to be structurally significantly flatter than trialkylamines without steric congestion, which is also signalized by the smaller heights of the NC_3_ pyramids (0.241–0.259 Å). There is no clear correlation between the heights of these pyramids and the degree of the steric crowding in the new amines, presumably because steric repulsion is partly compensated by dispersion interaction. In the cases of the two heterocyclic amines, the steric stress is smaller, and the molecular structures include quite different conformations. Quantum chemical calculations led to precise gas‐phase structures of the sterically overcrowded trialkylamines exhibiting heights of the NC_3_ pyramids and preferred molecular conformers which are similar to those resulting from the X‐ray studies.

## Graphical abstract

**Even record‐breaking steric stress** cannot enforce complete planarization of the nitrogen atom in trialkylamines as shown, for example, for di‐1‐adamantyl‐*exo*‐2‐norbornylamine with the help of single‐crystal X‐ray diffraction analysis and quantum chemical calculations in the solid phase as well as in the gas phase.

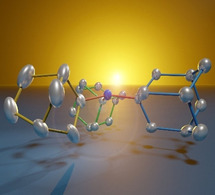

## Introduction

**Figure 1:**
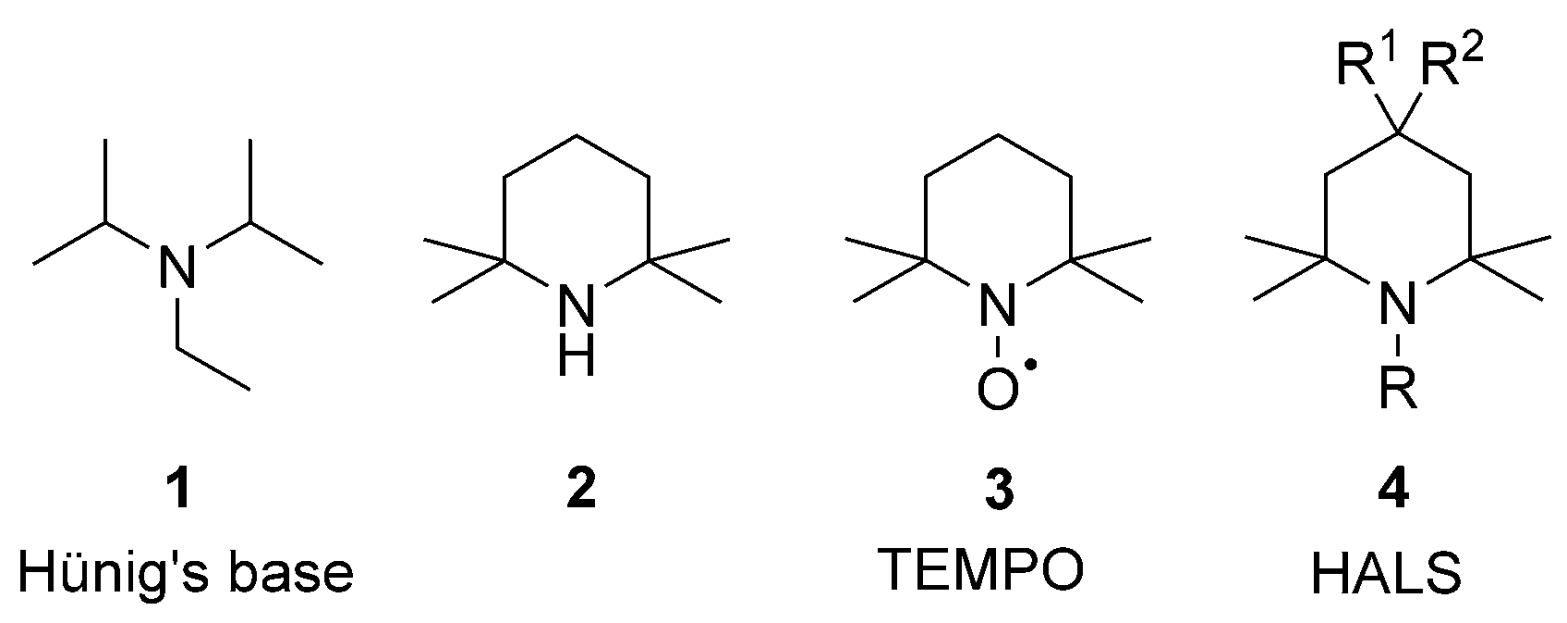
Sterically hindered amine derivatives with wide ranges of applications.

The amine entity belongs to the most important functional groups in chemistry.^[1]^ Quite distinct classes of amines are in the center of interest depending on the different properties and the respective fields of application. Tertiary and secondary amines with sterically demanding alkyl substituents, such as Hünig's base **1** ^[2]^ and 2,2,6,6‐tetramethylpiperidine (**2**),^[3]^ play a major role as Brønsted bases with low nucleophilicity or as precursors of persistent nitroxyl radicals, like **3**,^[4]^ which are used for spin labeling tools (Figure 1).^[5]^ Many heterocyclic compounds of type **4** and free radicals derived from these piperidines serve as polymerization inhibitors and photostabilizers, well known as hindered amine light stabilizers (HALS).^[6]^ Similar substances were recently introduced as base in frustrated Lewis pairs.^[7]^ Furthermore, amines with bulky alkyl groups were studied in view of their pharmacological activity.^[8]^ Finally, some sterically hindered amines have come into industrial use in gas‐treating processes.^[9]^

**Figure 2:**
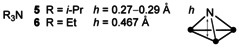
Pyramidal structures of amines **5** and **6** in the solid state.

In academia, other features than applications, such as new records of steric congestion^[10]^ and molecular structures of trialkylamines, are often in the focus of attention. Especially, the question whether amines with three bulky alkyl groups will adopt a planar instead of the pyramidal nitrogen atom to minimize the interaction of the amine substituents, has held a certain fascination for chemists.^[11]^ This question was mainly investigated by analyzing triisopropylamine (**5**) since other representatives with significantly higher steric distress were not accessible up to quite recently (Figure 2). Based on the results of electron diffraction studies, **5** should be very nearly planar about nitrogen with a value for the C−N−C bond angle of 119.2(3)°. Thus, a sum of the angles at the nitrogen atom of 357.6° was detected, which is quite close to 360°, an indication of a planar structure.^[12]^ This outcome was claimed to be confirmed by NMR investigations.^[13]^ However, low‐temperature single‐crystal X‐ray diffraction of **5** led to the C−N−C angle of 116.2(1)° and a sum of angles of 348.6°; thus a height of the NC_3_ pyramid (nitrogen at the top) of 0.27–0.29 Å (depending on temperature) was determined.^[14]^ The latter value is more than a half of the corresponding height in triethylamine (**6**), which amounts to 0.467 Å. In the solid state, the molecule of **5** obviously adopts a somewhat flatter pyramid instead of a planar structure,^[14]^ and this is significantly different to the corresponding results of the gas‐phase electron diffraction.^[12]^ It might be argued that crystal field effects are possibly responsible for the non‐planar molecular structure of **5** in a crystallized solid.

Herein, we describe the synthesis of several trialkylamines, which include significantly higher steric crowding than **5**. In the six cases of amines **8 a** and **8 e**–**i**, generation of single crystals and X‐ray diffraction were successful to analyze the molecular structures. Based on these results, quantum chemical calculations led to precise gas‐phase structures of the title compounds.

## Results and Discussion

### Synthesis of the amines

**Scheme 1:**
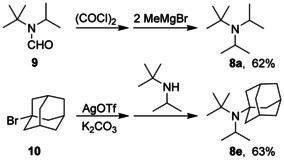
Synthesis of amines **8 a** and **8 e** by using alternative methods.

We mainly prepared tertiary amines **8**, in which steric distress surpasses that of the standard compound **5** distinctly, by treating *N*‐chlorodialkylamines **7** with Grignard reagents in the presence of a major excess of tetramethylethylenediamine (TMEDA).[^10a^, ^15^] The substrates **7** were easily available by chlorination of the corresponding secondary amines with the help of *N*‐chlorosuccinimide.^[16]^ Moderate yields of the alkylation products **8** were achieved as depicted in Table 1;^[17]^ however, in some cases, alternative methods led to better yields of the desired amines. For example, target compound **8 a** was also accessible by transforming formamide **9** into the respective chloroiminium chloride with the aid of oxalyl chloride, followed by the reaction with two equivalents of methylmagnesium bromide (Scheme 1). Furthermore, in situ generation of 1‐adamantyl triflate^[18]^ by exposure of bromide **10** to silver triflate and subsequent treatment with *tert*‐butylisopropylamine yielded the desired product **8 e**.^[19]^

**Table 1:** Synthesis of tertiary amines **8** from *N*‐chlorodialkylamines **7**.

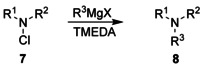				
	R^1^	R^2^	R^3^MgX	Yield of **8** [%]^[a]^
**a**	*t*‐Bu	*i*‐Pr	*i*‐PrMgCl	46
**b**	*t*‐Bu	*t*‐Bu	*i*‐PrMgCl	32
**c**	*t*‐Bu	*t*‐amyl	*i*‐PrMgCl	26
**d**	*t*‐amyl	*t*‐amyl	*i*‐PrMgCl	19
**e**	*t*‐Bu	1‐adamantyl	*i*‐PrMgCl	24
**f**	1‐adamantyl	1‐adamantyl	CyMgCl	25
**g**	1‐adamantyl	1‐adamantyl	norbornyl‐MgBr^[b]^	16
**h**	CMe_2_CH_2_C(OCH_2_CH_2_O)CH_2_CMe_2_		CyMgCl	18
**i**	CMe_2_CH_2_C(OCH_2_CH_2_O)CH_2_CMe_2_		*t*‐BuCH_2_MgBr	23
**j**	CMe_2_(CH_2_)_3_CMe_2_		*t*‐BuCH_2_MgBr	35

[a] Isolated yields. [b] 2‐*exo*‐Norbornyl Grignard reagent.

### Crystal and molecular structures of the amines

In the case of **8 a**,**b**,**d**,**j**, the title compounds proved to be colorless liquids at room temperature, whereas highly viscous liquids or waxy solids were obtained in other cases, and crystalline solids resulted by handling of **8 e**–**h** in methanol at different temperatures. Owing to the low melting points and the tendency to form plastic/disordered crystals, the crystallization and subsequent data collection as well as structure solution and refinement were very challenging for the compounds **8 a**, **8 e**, **8 f**, **8 g**, **8 h**, and **8 i**. Unfortunately, all attempts to obtain crystals which were suitable for structure determination with atomic resolution failed for compounds **8 b**, **8 c**, **8 d**, and the known^[20]^ model compound tri‐*tert*‐butylmethanol. The structure solution and refinement were performed using the programs SHELXS,^[21]^ SHELXT^[22]^ or Superflip^[23]^ (for structure solution) and SHELXL^[24]^ (for refinement) embedded in Olex2.^[25]^ Thus, single‐crystal X‐ray studies were successful for **8 a** and the five amines **8 e**–**i**, some crystallographic details are given in Table 2 (comprehensive data[^16^, ^26^]).

**Table 2:** Some crystallographic details of amines **8 a** and **8 e**–**i**.[^16^, ^26^]

	**8 a**	**8 e**	**8 f**	**8 g**	**8 h**	**8 i**
Empirical formula	C_10_H_23_N	C_17_H_31_N	C_26_H_41_N	C_27_H_41_N	C_17_H_31_NO_2_	C_16_H_31_NO_2_
Formula weight	157.29	249.43	367.60	379.61	281.43	269.42
Temperature [K]	100	100.0	100	100.00(13)	100	99.97(13)
Crystal system	monoclinic	monoclinic	monoclinic	monoclinic	monoclinic	monoclinic
Space group	*P*2_1_/*n*	*P*2_1_/*c*	*P*2_1_/*n*	*P*2_1_	*C*2/*m*	*P*2_1_/*c*
*a* [Å]	6.3045(10)	18.776(5)	16.46750(10)	6.4481(4)	14.812(7)	12.6810(15)
*b* [Å]	11.2957(17)	6.4752(18)	6.46350(10)	16.6531(7)	8.785(4)	22.441(2)
*c* [Å]	15.212(12)	24.315(6)	19.8240(2)	10.1178(6)	6.285(3)	12.6112(15)
*α* [°]	90	90	90	90	90	90
*β* [°]	97.947(2)	90.017(4)	102.7450(10)	105.420(6)	104.877(6)	116.987(15)
*γ*[°]	90	90	90	90	90	90
Volume [Å^3^]	1072.9(3)	2956.2(14)	2058.03(4)	1047.35(10)	790.3(6)	3198.0(7)
*Z*	4	8	4	2	2	8
*p_calc_* [g cm^−3^]	0.974	1.121	1.186	1.204	1.183	1.119
*μ* [mm^−1^]	0.056	0.063	0.493	0.502	0.076	0.072
*F(000)*	360.0	1120.0	816.0	420	312.0	1200.0
Crystal size [mm^2^]	0.6×0.3×0.3	0.711×0.216×0.086	0.168×0.129×0.074	0.155×0.046×0.029	0.404×0.403×0.24	0.5×0.5×0.5
Radiation *λ* [Å]	MoKα (*λ*=0.71073)	MoKα (*λ*=0.71073)	CuKα (*λ*=1.54184)	CuKα (*λ*=1.54184)	MoKα (*λ*=0.71073)	MoKα (*λ*=0.71073)
2*Θ* range for data collection [°]	4.506 to 62.936	1.674 to 50.816	6.33 to 160.578	9.066 to 136.488	5.44 to 62.98	3.604 to 54.202
Index ranges	−8≤h≤8, −16≤k≤15, −21≤l≤22	−22≤h≤22, −7≤k≤7, −29≤l≤29	−20≤h≤20, −8≤k≤7, −25≤l≤25	−6≤h≤7, −20≤k≤20, −12≤l≤12	−21≤h≤20, −12≤k≤12, −9≤l≤8	−16≤h≤16, −28≤k≤28, −16≤l≤16
Reflections collected	12 400	33 220	23 2051	3721	4638	54 266
Independent reflections	3276 [*R_int_=*0.0248, *R_sigma_*=0.0215]	5434 [*R_int_=*0.0607, *R_sigma_*=0.0371]	4451 [*R_int_=*0.0756, *R_sigma_*=0.0128]	3721 [*R_int_=*0.095, *R_sigma_*=0.0122]	1297 [*R_int_=*0.0360, *R_sigma_*=0.0367]	7051 [*R_int_=*0.0686, *R_sigma_*=0.0478]
Data/restraints/ parameters	3276/0/192	5434/0/336	4451/0/409	3721/1/254	1297/182/171	7051/0/357
Goodness‐of‐fit on *F* ^*2*^	1.072	1.034	1.114	1.048	1.060	1.026
Final *R* indexes [*I*>=2° (*I*)]	*R1=*0.0539, *wR2=*0.1395	*R1=*0.0435, *wR2=*0.0987	*R1=*0.0579, *wR2=*0.1342	*R1=*0.0878, *wR2=*0.2420	*R1=*0.0495, *wR2=*0.1266	*R1=*0.0548, *wR2=*0.1147
Final *R* indexes [all data]	*R1=*0.0594, *wR2=*0.1447	*R1=*0.0556, *wR2=*0.1044	*R1=*0.0585, *wR2=*0.1346	*R1=*0.0928, *wR2=*0.2480	*R1=*0.0799, *wR2=*0.1418	*R1=*0.0830, *wR2=*0.1284
Largest diff. peak/hole [e Å^−3^]	0.56/−0.20	0.26/−0.19	0.38/−0.24	1.04/−0.24	0.10/−0.16	0.23/−0.22
CCDC^[26]^	2002567	2002569	2002571	2002570	2002568	2004218

**Figure 3:**
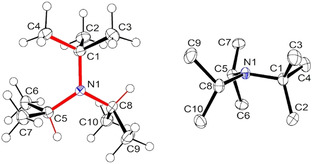
Molecular structure of amine **8 a** as determined from the crystal structure analysis; the triskele‐like conformation is emphasized by red color. The ellipsoids are shown at the 50 % probability level.^[27]^

**Figure 4:**
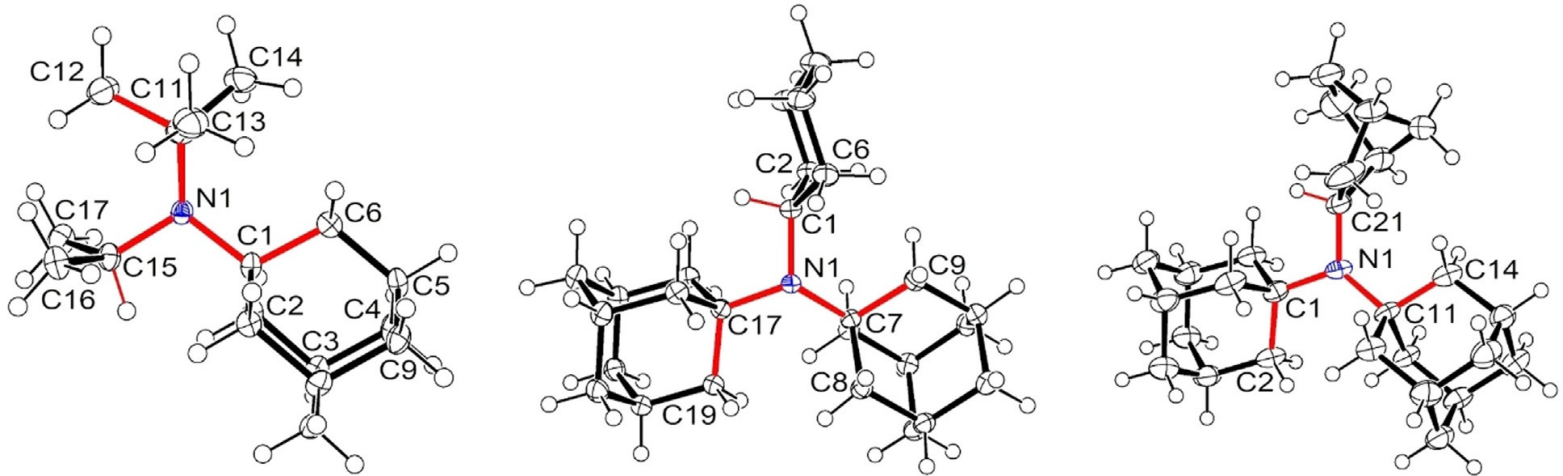
Molecular structures of the amines **8 e** (left), **8 f**, and **8 g** (right) in the solid state; the triskele‐like conformations are emphasized by red color. All ellipsoids are shown at the 50 % probability level.^[27]^

The molecular structure of amine **8 a** in the single crystal shows a triskele‐like conformation with three arms around the nitrogen atom [N1‐C1‐C4, N1‐C5‐H(C5), and N1‐C8‐H(C8), see Figure 3], which obviously enables optimal packing of the bulky alkyl groups. Similar triskele‐like conformations were also detected in the case of the three other noncyclic amines **8 e**, **8 f**, and **8 g** (Figure 4). These preferred conformations can be characterized by selected, roughly antiperiplanar torsion angles as depicted in Table 3. When the C−N bond lengths of **8 a** are compared with those of the sterically more stressed amines **8 e**, **8 f**, and **8 g**, slightly greater values are found in the latter cases, even for C−N bonds connecting the same alkyl group with nitrogen; for example, *t*Bu‐N in **8 a** leads to a bond length of 1.4786(10) Å, whereas **8 e** revealed 1.491(3) Å. The greatest C−N distances were always experimentally observed for the nitrogen‐attached 1‐adamantyl units (1.50–1.52 Å). The C−N−C bond angles ranged from 109.7 to 123.2° (Table 3). As expected, a small angle was detected for the *i*Pr−N−*i*Pr group in **8 a** [113.17(6)°], while the greatest angle value resulted for amine **8 f**, in which nitrogen is bridging two 1‐adamantyl moieties. However, two quite different C−N−C angles were found in single amines even if the nitrogen is connected with a pair of the same alkyl groups. For example, the molecular structure of **8 f** includes two C_1‐adamantyl_−N−C_cyclohexyl_ angles of 109.70(13) and 119.49(13)°; in the case of the smaller angle, the H(C1)−C1 bond (triskele arm) points to a 1‐adamantyl unit, and obviously this is sterically more favorable than the interaction of C2 and C6 of the cyclohexyl group with the C7–C9 moiety (another triskele arm) of the other 1‐adamantyl substituent (Figure 4). A similar situation was detected in the molecular structure of **8 g** indicating two rather distinct C_1‐adamantyl_−N−C_2‐norbornyl_ angles of 111.1(4) and 120.4(4)°.

**Table 3:** Some molecular details of amines **8 a** and **8 e–i** resulting from X‐ray studies.[^16^, ^26^]

	**8 a**	**8 e^[a]^**	**8 f**	**8 g**	**8 h**	**8 i^[b]^**
C−N bond lengths [Å]	1.4786(10) 1.4757(10) 1.473910)	1.516(3) 1.491(3) 1.481(3)	1.490(2) 1.500(2) 1.518(2)	1.501(7) 1.508(6) 1.484(7)	1.492(4) 1.486(3) 1.485(3)	1.4751(19) 1.498(2) 1.493(2)
Bond angles at nitrogen [°]	122.77(6) 115.60(6) 113.17(6)	122.86(17) 110.57(15) 117.71(18)	119.49(13) 109.70(13) 123.17(13)	120.8(4) 111.1(4) 120.4(4)	110.6(7) 119.2(2) 120.6(7)	113.96(12) 114.15(12) 116.60(12)
Sum of the three angles at nitrogen [°]	351.54	351.14 351.20	352.36	352.3	350.4	344.71 344.37
Height of the NC_3_ pyramid (nitrogen at the top) [Å]	0.2537(3)	0.2588(19) 0.2585(19)	0.2410(18)	0.243(5)	0.268(3)	0.3414(16) 0.3450(17)
Selected torsion angles [°]	C1‐N1‐C5‐H(C5) 166.0(7) C5‐N1‐C8‐H(C8) 165.5(7) C8‐N1‐C1‐C4 −177.45(7)	C1‐N1‐C11‐C12 178.20(16) C11‐N1‐C15‐H(C15) −162.65(1) C15‐N1‐C1‐C6 −176.79(16)	C1‐N1‐C17‐C19 176.20(14) C7‐N1‐C1‐H(C1) 164.8(12) C17‐N1‐C7‐C9 179.30(15)	C1‐N1‐C11‐C14 −173.7(4) C11‐N1‐C21‐H(C21) −163.7(5) C21‐N1‐C1‐C2 178.0(5)	N1‐C1‐C2‐C3 167.4(6) N1‐C1‐C6‐C5 −171.0(6) C1‐N1‐C7‐C8 171.0(6) C1‐N1‐C11‐C10 −162.3(7)	C1‐N1‐C6‐C9 −169.93(13) C1‐N1‐C12‐C11 169.08(13)

[a] A second molecule in the asymmetric unit leads to another set of C−N bond lengths with 1.497(3), 1.497(3), and 1.501(3) Å and another set of bond angles at nitrogen with 122.11(7), 110.00(16), and 119.09(17)° as well as another set of the corresponding torsion angles.^[16]^ [b] A second molecule in the asymmetric unit leads to another set of C−N bond lengths with 1.475(2), 1.490(2), and 1.499(2) Å and another set of bond angles at nitrogen with 114.16(12), 113.56(13), and 116.65(12)° as well as another set of the corresponding torsion angles.^[16]^

Even though the C−N−C bond angles vary significantly, the sum of the three angles at the nitrogen atom is always in the range of 351.1 to 352.4° in the cases of the noncyclic amines **8 a**, **8 e**, **8 f**, and **8 g**. Hence, the four amines proved to be structurally flatter than triisopropylamine (**5**), which is also demonstrated by the smaller heights of the NC_3_ pyramids (0.241–0.259 Å) as shown in Table 3. However, there is obviously no clear correlation between the heights of these pyramids and the increasing degree of the steric stress in the order **8 a**, **8 e**, **8 f**, **8 g**. This result may lead to the assumption that there is a limit in the height of NC_3_ pyramids, which cannot be significantly smaller than 0.24 Å even in the case of sterically overcrowded trialkylamines.

Since product 8 g was prepared from **7** and an equilibrating mixture of *exo*‐ and *endo*‐2‐norbornylmagnesium bromide^[28]^ (Table 1), an alternative stereoisomeric structure of this tertiary amine including an *endo*‐2‐norbornyl group was also thinkable. Thus, the X‐ray crystal structure analysis confirmed now the *exo*‐2‐norbornyl structure of 8 g, which was previously assigned by NMR spectroscopy.^[10a]^ Recently, two non‐equivalent rotamers of **8 e** were detected in a 5:1 ratio using high resolution NMR methods. Because this amine bears three different bulky alkyl groups, it obviously is able to adopt two distinct triskele‐like conformations in solution.^[10a]^ The main rotamer in solution corresponds to the molecular structure of 8 e in the single crystal.

**Figure 5:**
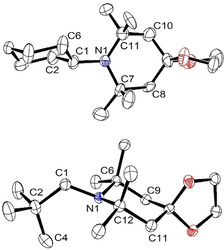
Molecular structures of the amines **8 h** (top) and **8 i**. All ellipsoids are shown at the 50 % probability level.^[27]^ Hydrogen atoms are omitted for clarity.

In the molecular structures of the heterocyclic amines **8 h** and **8 i**, determined by the X‐ray crystal structure analysis, the piperidine rings and also the cyclohexane ring of **8 h** adopt chair conformations (Figure 5). The amino group at the cyclohexane moiety of **8 h** is in an equatorial position, and the same is true for the cyclohexyl and the neopentyl groups at the piperidine units of **8 h** and **8 i**, respectively. These equatorial positions are confirmed by roughly antiperiplanar torsion angles as depicted in Table 3. However, the conformations of the exocyclic substituents at the nitrogen atoms are quite different: Whereas H(C1) of the cyclohexyl group points to C7 and the angles C1‐N1‐C7 [110.6(7)°] and C1‐N1‐C11 [120.4(4)°] are rather distinct in **8 h**, the molecular structure of **8 i** is more symmetric with similar angles C1‐N1‐C6 [113.96(12)°] and C1‐N1‐C12 [114.15(12)°] as well as similar absolute values of the torsion angles C2‐C1‐N1‐C6 [111.14(16)°] and C2‐C1‐N1‐C12 [−111.44(16)°]; furthermore, torsion angle N1‐C1‐C2‐C4 [4.7(2)°] is very small.

Although amine **8 h** bears a secondary and two tertiary alkyl groups at the nitrogen, the steric stress is smaller than that of **8 e**, **8 f**, and **8 g** because of the piperidine ring structure, which connects both tertiary alkyl moieties in **8 h**. Consequently, the sum of the three angles at the nitrogen atom of **8 h** is slightly smaller and the height of the NC_3_ pyramid is somewhat greater than the corresponding values of amine **8 a** that includes two secondary and only a single tertiary alkyl group at nitrogen (Table 3). In the case of the compound **8 i** with a primary alkyl unit at the piperidine N‐atom, the sum of the three C−N−C angles proves to be significantly smaller than that of triisopropylamine (**5**); and the height of the NC_3_ pyramid is considerably greater than that of **5**.

We do not believe that crystal field effects are responsible for the non‐planar molecular structures of our sterically overcrowded trialkylamines. In order to confirm this assumption, structural characterization of these amines in the gas phase, based on high‐quality quantum chemical calculations, will be helpful.

### Quantum chemical calculations

In order to elucidate the effect of crystallization further, we did detailed calculations on all the species in the gas as well as the solid crystalline phase. Furthermore, we computed other similar compounds with smaller alkyl groups. Finally, we did a conformational search of all the systems **8 a**–**8 j**, in order to see if the conformer in the periodic crystal indeed corresponds to the minimum structure found in the gas phase.

The effect of different generalized gradient approximation (GGA) density functionals is also tested. In Table 4, we compare the experimental crystal structures to the experimental one and its gas phase structures.

**Table 4:** Effect of different methods and the environment on the molecular structures **8 a**, **8 e**, **8 f**, **8 g**, **8 h**, and **8 i** on the height of the NC_3_ pyramid (nitrogen at the top) in Å.

	exp.	DFT average^[a]^		BLYP^[30]^+D3^[31]^		PBE^[32]^+D3	
		crystal	gas	crystal	gas	crystal	gas
**8 a**	0.254	0.243	0.230	0.245	0.230	0.245	0.232
**8 e**	0.259	0.254	0.242	0.258	0.241	0.250	0.240
**8 f**	0.241	0.226	0.247	0.223	0.248	0.228	0.246
**8 g**	0.243	0.234	0.236	0.232	0.237	0.232	0.234
**8 h**	0.268	0.253	0.243	0.254	0.239	0.251	0.240
**8 i**	0.341	0.318	0.329	0.316	0.325	0.319	0.328

[a] Average of the BLYP^[29]^+D3,^[30]^ optB88‐vdW,^[31]^ PBE^[32]^+D3, PBE+TS,^[33]^ RPBE^[34]^+D3 and vdW‐DF2^[35]^ GGA functionals.

Since different GGA functionals, like BLYP, PBE, and even the average of our computed methods yield very similar results, we do not believe that these will change when using another, different method. The computed crystal structures have a lower height of the NC_3_ pyramid than the experimental ones, probably due to temperature and zero‐point effects on the cell volume. And whereas the height is even lower in the gas phase of the less crowded compounds **8 a**, **8 e**, and **8 h**, it becomes larger for the gas phase of **8 f**, **8 g**, and **8 i**. Still, all of the reported heights are in the range of 0.225–0.350 Å, indicating that this is the value to be expected for such compounds.

Less hindered trialkylamines, such as trimethylamine, usually exhibit larger NC_3_ heights. For triethylamine (**6**) and tripropylamine, different conformers will of course give different values for the heights. This is illustrated in Table 5, where the pyramidal NC_3_ height can also vary widely between 0.3 and 0.45 Å. In order to discuss not only the pyramidal heights, but also the energies needed to make the molecule more planar, we enforced the NC_3_ substructure to be in one plane; we set the dihedral angles to zero regarding all CNC planes and reoptimized the minimum configurations. Although this is not necessarily the transition state on the potential energy surface since all side groups will have to invert as well, it yields an estimate of the umbrella motion for the minimum structure to become planar. For trimethylamine, the B3LYP^[36]^+D3/TZVPPD^[37]^ barrier is rather high with as much as 31.2 kJ mol^−1^, whereas it is lowered to 15 kJ mol^−1^ for triethylamine and 15.9 kJ mol^−1^ for tri‐*n*‐propylamine. The optimized B3LYP/TZVPPD structures without dispersion yield 29.1, 17.9, and 15.2 kJ mol^−1^, respectively, implying that the barrier for trimethylamine and tripropylamine are somewhat lowered by van der Waals interactions, whereas for triethylamine, it is larger.

**Table 5:** Different gas phase conformers of tertiary amines R_3_N with their pyramidal NC_3_ heights in Å using B3LYP+D3/TZVPPD. The energy differences to the lowest conformer are given in kJ mol^−1^.

R	methyl	ethyl		*n*‐propyl		isopropyl	
	NC_3_	Energy	NC_3_	Energy	NC_3_	Energy	NC_3_
1	0.430	0	0.424	0	0.411	0	0.200
2		2.1	0.423	1.9	0.415	15.3	0.330
3		3.2	0.388	3.2	0.419	15.7	0.211
4		7.5	0.301	4.4	0.373	25.9	0.287
5				4.6	0.370

Triisopropylamine (**5**), which has been previously mentioned and investigated more than 20 years ago, is a particular interesting case: A similar analysis between the planar and the non‐planar structure in the gas phase of this compound yields an extremely low inversion barrier of only 2.4 kJ mol^−1^ and a pyramidal height of only 0.200 Å for B3LYP+D3/TZVPPD. Whereas basis set limit CCSD(T)^[16]^ increases this barrier to 4.8–5.3 kJ mol^−1^ depending on the geometry used, the zero‐point energy contribution lowers the barrier by 3.0 kJ mol^−1^. Thus, including the zero‐point energy contribution and using more accurate post‐Hartree–Fock methods, we would end up around 1.8–2.3 kJ mol^−1^ energy difference between the planar and the non‐planar structure. The transition state has an extremely small imaginary frequency of 88 cm^−1^ when using B3LYP+D3,

When neglecting dispersion, the value of 2.4 kJ mol^−1^ decreases the B3LYP barrier by 1.4 kJ mol^−1^ to 1.0 kJ mol^−1^. Since in a molecular crystal, the dispersion is more uniform than for a single molecule, this decrease may explain that we obtain an almost planar structure with an height of 0.03 Å, rather independent on the functional used when reoptimizing the solid crystal structure of this compound which has been reported as disordered with a pyramidal height of 0.291 Å.^[14]^

Interestingly, the calculations provide exactly the opposite results than experiment, in which the gas phase structure was determined to be planar,^[12]^ whereas the crystal structure was non‐planar: For the gas phase, this is likely due to the extremely flat potential energy surface around the minimum structure. For the solid phase, we were not able to discern the exact cause of this discrepancy between experiment and theory: It is not the thermal and zero‐point expansion of the cell volume, as larger cell volumes yield similar planar structures. The culprit for these differences are either the underestimation of DFT for the barrier in general or thermal motions which are not easily described by and modelled by theory.

**Figure 6:**
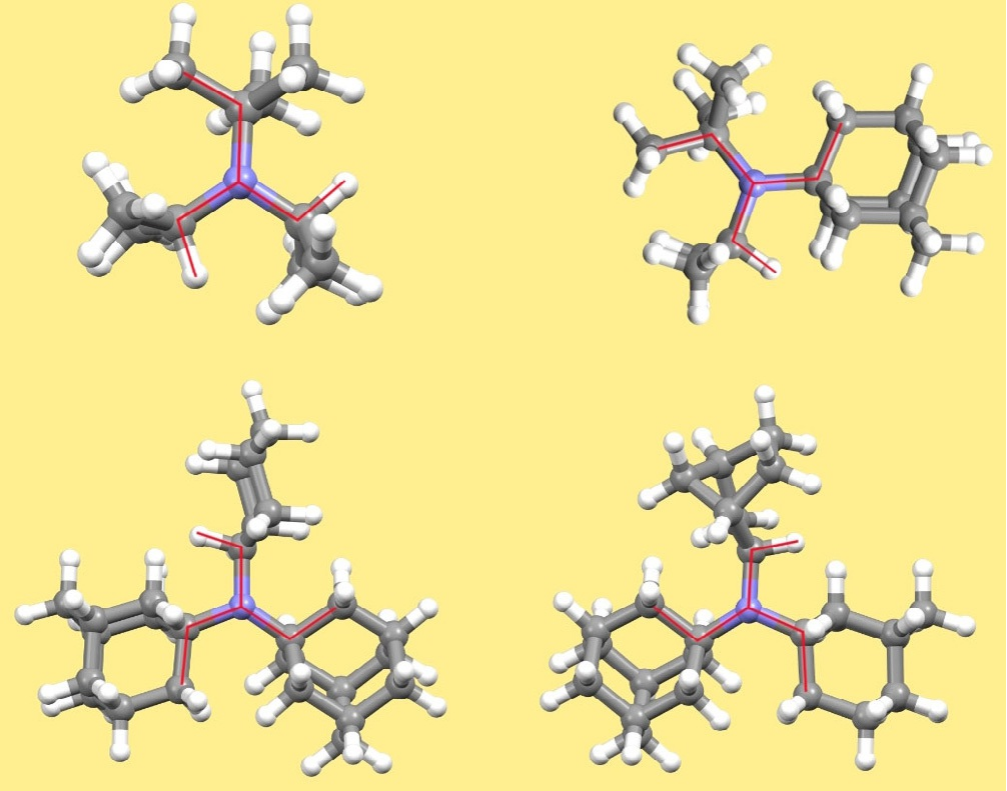
Lowest energy structures of amines **8 a**, **8 e**, **8 f**, and **8 g** in the gas phase calculated using B3LYP+D3/TZVPPD. The triskele‐like conformations are emphasized by red color.

Continuing with compounds **8 a**–**8 j** synthesized, a similar analysis like in Table 5 is performed in Table 6, whereas an analysis of **8 g** and **8 i** only gives one conformer within a given energy range of 30 kJ mol^−1^. It is important to note that in all cases, the lowest energy structure of the gas phase is the one also found in the crystalline phase, see Figures 3, Figure 4, and Figure 6 with structures of **8 a**, **8 e**, **8 f**, and **8 g**. Perhaps contrary to initial intuition, the barriers to planarity and the pyramidal NC_3_ heights increase again after being rather small for compound **8 a**. This is an effect to the van der Waals interactions of the large, bulky groups, which attract each other: When optimizing all structures without extra dispersion, using just B3LYP/TZVPPD (without D3 correction), all barriers of the more bulky compounds **8 a**–**8 j** investigated are lowered and all pyramidal heights are consequently lowered.

**Table 6:** Gas phase conformer effects on the pyramidal NC_3_ heights in Å using B3LYP+D3/TZVPPD of the five lowest conformers of the compounds **8 a‐**‐**8 j**. The second row **E** is the same as the column “Energy” in Table 5, displaying the energy difference to the lowest conformer in kJ mol^−1^.

	**8 a**	**8 b**	**8 c**	**8 d**	**8 e**	**8 f**	**8 h**	**8 j**
1	0.219	0.229	0.226	0.256	0.231	0.233	0.223	0.309
**E_p_** ^**[a]**^	6.1	13.3	10.5	10.4	10.2			24.2
2	0.202	0.07	0.228	0.214	0.202	0.092	0.086	0.316
**E**	4.0	11.8	0.4	1.3	4.4	9.7	6.9	0.5
3	0.149	0.210	0.214	0.239	0.238	0.216	0.212	0.353
**E**	26.3	22.2	5.3	3.9	4.4	24.6	10.2	4.4
4			0.225	0.214	0.075		0.104	0.207
**E**			7.1	4.7	12.5		23.3	12.7
5			0.217	0.204	0.100			0.292
**E**			8.1	8.3	13.0			16.0

[a] Energy difference of the lowest energy conformer 1 and when reoptimizing this configuration with its pyramidal height set to zero by defining the dihedral angles regarding all CNC planes to zero.

In general, the conformational structure seems to have a very large effect on the planarity. For example, compound **8 e** has one almost planar structure when being in a conformer which is about 13 kJ mol^−1^ above the gas phase minimum structure, and **8 f** one conformer which is about 10 kJ mol^−1^ above the gas phase minimum. In case a crystal structure could trap one of these conformers in a polymorph, we would obtain an NC_3_ height close to zero. Overall, this effect thus shows structures (in the gas phase) in a much wider range than the different crystal structures of the synthesized compounds, ranging between 0.08 and 0.33 Å for the NC_3_ heights.

## Conclusions

In summary, the molecular structures of our sterically overcrowded trialkylamines, which do not include any π system or hetero atom in proximity to the nitrogen atom, proved to be pyramidal with NC_3_ heights that are significantly smaller than those of simple species such as trimethylamine or triethylamine. Tertiary amines with heteroatom functionalities in the α or β positions were previously investigated, also by using X‐ray studies, and led to nearly planar molecular structures, which were explained by orbital interaction effects.^[38]^ In our cases of trialkylamines, steric effects alone obviously cannot enforce complete planarization of the amine nitrogen. Crystal field effects are not responsible for the non‐planar structures of such trialkylamines because characterization in the gas phase, based on high‐quality quantum chemical calculations, led to structural results which are similar to those of single‐crystal X‐ray diffraction analysis. On the one hand, van der Waals interactions of the bulky alkyl groups are a plausible explanation that even record‐breaking steric stress cannot enforce complete planarization of the nitrogen atom. On the other hand, dispersion^[39]^ plays also a role in the molecular structures of the title compounds.

As shown by our X‐ray studies as well as quantum chemical calculations for the molecules in the gas phase, the noncyclic amines **8 a** and **8 e**‐**g** adopt triskele‐like conformations, which can be utilized to interpret the corresponding temperature‐dependent high‐resolution NMR spectra. The same is true for the quite different conformations of the heterocyclic amines **8 h** and **8 i**. Currently, we are investigating rotation processes within these amines with the help of dynamic NMR spectroscopy. Furthermore, we are trying to prepare tertiary amines with even more steric crowding, for example, open‐chain tri‐*tert*‐alkylamines, by oxidative ring opening of unsaturated 2,2,6,6‐tetramethylpiperidines and 2,2,5,5‐tetramethylpyrrolidines.

## Conflict of interest

The authors declare no conflict of interest.

## Supporting information

As a service to our authors and readers, this journal provides supporting information supplied by the authors. Such materials are peer reviewed and may be re‐organized for online delivery, but are not copy‐edited or typeset. Technical support issues arising from supporting information (other than missing files) should be addressed to the authors.

Supplementary
