# Supplementary material for: Non‐Planar Structures of Sterically Overcrowded Trialkylamines
Source: Chemistry. 2020 Dec 14;27(11):3700–7. doi: 10.1002/chem.202003933 (PMC7984391; doi:10.1002/chem.202003933)
Supplement: Supplementary file 1 — Supplementary [file CHEM-27-3700-s001.pdf]

Chemistry–A European Journal

Supporting Information

Non-Planar Structures of Sterically Overcrowded Trialkylamines

Klaus Banert†,*[a] Manuel Heck,[a] Andreas Ihle,[a] Tharallah Shoker,[a] Michael Wörle,*[b] and A. Daniel Boese*[c]

Supporting Information
©Wiley-VCH 2020
69451 Weinheim, Germany

DOI: 10.1002/chem.2020XXXXX

[a] Prof. Dr. K. Banert, M. Heck, Dr. A. Ihle, Dr. T. Shoker
Organic Chemistry, Chemnitz University of Technology
Strasse der Nationen 62, 09111 Chemnitz (Germany)
E-mail: klaus.banert@chemie.tu-chemnitz.de

[b] Dr. M. Wörle
Department of Chemistry and Applied Biosciences, ETH Zürich
Vladimir-Prelog-Weg 1, 8093 Zürich (Switzerland)
E-mail: michael.woerle@inorg.chem.ethz.ch

[c] Prof. A. D. Boese
Institute of Chemistry, Physical and Theoretical Chemistry,
University of Graz
Heinrichstrasse 28/IV, 8010 Graz (Austria)
E-mail: Adrian_Daniel.Boese@uni-graz.at

Table of Contents

Experimental and Theoretical Procedures....................S-2

Printed NMR-Spektra....................S-5

Crystal Structures....................S-13

Theoretical Data....................S-51

References....................S-59

Experimental and Theoretical Procedures

General Methods: Melting points were determined with a Pentakon Dresden Boetius apparatus. ^{1}H and ^{13}C NMR spectra were recorded with Bruker Avance HD 600 spectrometer operating at 600 MHz and 151 MHz, respectively. Chemical shifts δ are given in ppm and were referenced to the solvent signals ($CDCl_3$: 7.26 ppm (^{1}H), 77.0 ppm (^{13}C), C_6D_6: 7.16 ppm (^{1}H), 128.0 ppm (^{13}C)).The multiplicities of ^{13}C NMR signals were determined with the aid of HSQC or HMBC experiments. The multiplicities were described using the following abbreviations and their combinations: s (singlet), d (doublet), t (triplet), q (quartet), m (multiplet).^{1}H NMR yields were measured with solvent as internal standard. HRMS (ESI) spectra were recorded with a 15 T solariX FT-ICR-MS or a Bruker micrOTOF QII spectrometer.

Single crystal X-ray diffraction analyses: Single crystals of the amines were either crystallized from ethanol or methanol and subsequently tip-mounted using Mitegen micromounts or grown on the diffractometer in Mark-tubes by slow cooling and/or tempering of the liquid amine. The crystals were kept at 100 K during data collection. Using Olex2 [S-1], the structures were solved with the SHELXS [S-2], ShelXT [S-3] or Superflip [S-4] structure solution programs and refined with the ShelXL [S-5] refinement package using Least Squares minimisation. Experimental details of the crystal growth and the structure analyses for each amine are given in the respective sections below.

Theoretical Methods employed: For the gas phase calculations, we employed TURBOMOLE 7.4 [S-6] and for the periodic solid phase calculations VASP 5.4.1. [S-7]

For the latter, we used a 900 eV energy cut-off and hard pseudopotentials (for optB88-vdW and vdW-DF02, we used a 1200 eV cut-off). The convergence criteria for the periodic structure relaxation correspond to 10^{-5} eV for the energy and to 5×10^{-3} eV Å^{-1} for the gradient. For BLYP+D3, we furthermore tested a somewhat larger cut-off energy of 1200 eV and found very little variance in the geometry (see Tables S-25 and S-26).

All optimizations were carried out using a k-point grid according to the lengths of the cell dimensions of the respective compounds: 3x2x1 for compound **8a**, 2x4x1 for compound **8e**, 1x3x1 for compound **8f**, 3x1x2 for compound **8g**, 2x2x3 for compound **8h**, 2x1x2 for compound **8g**, 3x4x3 for trimethylamine and 2x2x1 for triisopropylamine.

When directly comparing gas phase calculations with the optimized crystal structures in Table 4, we compute all monomers of the GGA functionals in a box of at least 17x17x17 Ångstrom up to 22x22x22 Ångstrom depending on the size of the molecule, turning off dispersion beyond a radius of 10 Ångstrom in order to prevent the molecules of the different boxes to interact with each other. In some example cases, we tested the convergence of the lattice energy (which is much more susceptible to the monomer cell size than the geometry) by going to larger boxes, finding no difference between these.

For the post-Hartree-Fock gas phase calculations like the MP2, SCS-MP2 optimizations of Table S-25 and the CCSD(T) calculations, we extrapolated Dunning's aug-cc-pVXYZ basis sets (with XY ranging from double-ζ to quintuple-ζ quality).[S-8] This has been done with the X^{-3} formula for the correlation contribution[S-9] and with the X^{-5} formula for Hartree-Fock [S-10]. For MP2 and SCS-MP2, we optimized the structures of Table S-25 including the extrapolation formula, whereas for the CCSD(T) single-points of triisopropylamine, we utilized the B3LYP+D3/TZVPPD and the MP2/aug-cc-pV(D,T)Z extrapolated structures.

Syntheses:

The *N*-chloroamines were prepared according to the literature.[S-11] The compounds **8a**-**g** were synthesized by methods which were described in the literature.[S-12] **8c**, for example, was purified by preparative gas chromatography (column: Carbowax + KOH, 1 m, oven: 90 °C, det.: 100 °C, He: 30 mL/min) and isolated as a white solid. M.p.: 67 °C.

Synthesis of 8-chloro-7,7,9,9-tetramethyl-1,4-dioxa-8-azaspiro[4.5]decane (7h)

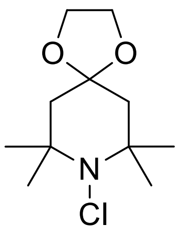

The secondary amine 7,7,9,9-tetramethyl-1,4-dioxa-8-azaspiro[4.5]decane [S-13] (10 mmol) is dissolved into abs. DCM (10 mL), and *N*-chlorosuccinimide (11 mmol) is slowly added. The mixture is stirred for two hours at rt. Then pentane (20 mL) is added and the organic phase is washed with water (3 x 25 mL). After drying over K_2CO_3 and removing the solvent, the product can be isolated with a yield of 2.2 g (9.4 mmol, 94%).

^{1}H NMR [400 MHz, $CDCl_3$] δ (ppm) 1.31 (s, 12H, CH_3), 1.83 (s, 4H, C-CH_2), 3.91 (s, 4H, O-CH_2).

^{13}C NMR [100.6 MHz, $CDCl_3$] δ (ppm) 27.6 (q, CH_3), 46.8 (t, C-CH_2), 62.5 (t, O-CH_2), 63.8 (s, N-C), 106.4 (s, O-C-O).

HRMS: $C_{11}H_{20}ClNNaO_2$ $[M + Na]^+$ calc.: 256.107477, exp.: 256.10748.

Procedure A: TMEDA (6 mmol) and the respective organometallic alkylation reagent (3 mmol) are mixed in anhydrous Et_2O or THF (10 mL) at −78 °C and stirred for one hour. The chloroamine **7** (1 mmol) is added to Et_2O/THF (5 mL) and the solution is slowly added dropwise. The mixture is stirred for two hours and allowed to warm to room temperature. Then the reaction mixture is put on ice/water (100 mL) and extracted with Et_2O (3 x 50 mL). The organic phase is washed with water (5 x 30 mL) and dried with K_2CO_3. The solvent is removed under vacuum and the residue chromatographically purified over basic alumina (Et_2O:hexane). In some cases the undesired secondary amine has to be condensed off separately at a vacuum of 10^{-3} mbar.

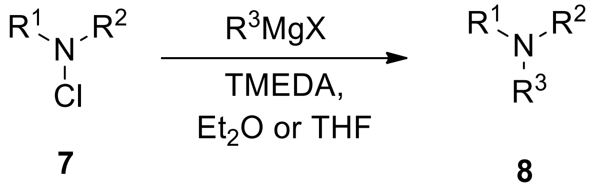

8-Cyclohexyl-7,7,9,9-tetramethyl-1,4-dioxa-8-azaspiro[4.5]decane (8h)

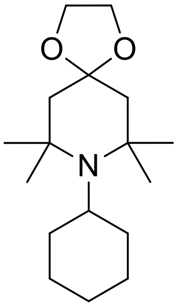

According to procedure A, CyMgCl (3 mL, 6 mmol, 2M in Et_2O) and 8-chloro-7,7,9,9-tetramethyl-1,4-dioxa-8-azaspiro[4.5]decane (**7h**, 500 mg, 2.13 mmol) are used. Thus the product can be isolated by condensing out the sec. amine at 10^{-2} mbar with a yield of 105 mg (0.373 mol, 18%). Furthermore, it could be recrystallized from methanol or ethanol at rt.

mp.: 90–95° C.

^{1}H NMR [600 MHz, C_6D_6] δ (ppm) 1.01 (dtt, ^2J = 13.1 Hz, ^3J = 13.1 Hz, 3.6 Hz, 1H, CH_2), 1.20 (dtt, ^2J = 13.8 Hz, ^3J = 13.8 Hz, 4.2 Hz, 2H, CH_2), 1.29 (bs, 6H, C-CH_3), 1.40 (bs, 6H, C-CH_3), 1.55--1.57 (m, 1H, CH_2), 1.67--1.74 (m, 4H, CH_2), 1.79--1.84 (m, 6H, CH_2), 2.98 (tt, ^3J = 11.9 Hz, 2.7 Hz, 1H, CH), 3.54 (s, 4H, O-CH_2-CH_2).

^{13}C NMR [151 MHz, C_6D_6] δ (ppm) 26.7 (t, CH_2), 28.5 (t, CH_2), 30.5 (q, CH_3), 32.1 (q, CH_3), 37.9 (t, CH_2), 48.9 (t, O-C-$\underline{C}H_2$), 52.6 (t, O-C-$\underline{C}H_2$), 56.1 (s, N-C), 57.3 (s, N-C), 57.7 (d, CH), 63.7 (t, O-CH_2), 107.9 (s, O-C-O).

HRMS: $C_{17}H_{32}NO_2$ $[M + H]^+$ calc.: 282.2428, exp.: 282.2435.

8-Neopentyl-7,7,9,9-tetramethyl-1,4-dioxa-8-aza-spiro[4.5]decan (8i)

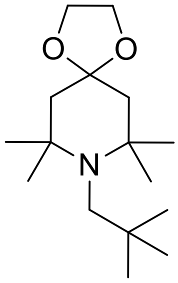

The synthesis is carried out analogously to procedure A with 8-chloro-7,7,9,9-tetramethyl-1,4-dioxa-8-aza-spiro[4.5]decane (**7h**, 2 mmol, 460 mg) and neopentMgBr (4.5 mmol, 1 M in Et_2O). The product can be obtained as a viscous liquid with a yield of 126 mg (0.47 mmol, 23%).

mp.: −2 °C.

^{1}H NMR [600 MHz, C_6D_6] δ (ppm) 1.01 (s, 9H, *t*Bu), 1.06 (s, 6H, 7/9-Me), 1.25 (s, 6H, 7/9-Me), 1.71 (d, ^2J = 13.0 Hz, 2H, 6/10-CH_2), 1.89 (d, ^2J = 13.2 Hz, 2H, 6/10-CH_2), 2.46 (s, 2H, N-CH_2), 3.49 (t, J = 6.3 Hz, 2H, O-CH_2), 3.56 (t, J = 6.3 Hz, 2H, O-CH_2).

^{13}C NMR [151 MHz, C_6D_6] δ (ppm) 22.0 (q, 7/9-Me), 30.9 (s, *t*Bu), 31.3 (q, *t*Bu), 36.8 (q, 7/9-Me), 48.7 (t, C-6/10), 55.0 (s, N-C), 56.0 (t, N-CH_2), 62.7 (t, O-CH_2), 64.6 (t, O-CH_2), 107.9 (s, O-C-O).

HRMS: $C_{16}H_{32}NO_2$: $[M + H]^+$ calc.: 270.2428, exp.: 270.2429.

***N*-Neopentyl-2,2,6,6-tetramethylpiperidine (8j)**

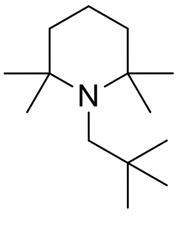

Procedure A: With neopentylmagnesium bromide (4.5 mmol, 1 M in Et_2O) and *N*-chloro-2,2,6,6-tetramethylpiperidine [S-14] (**7j**, 2 mmol, 350 mg) in use, the product can be obtained as a colorless liquid after recondensing at 10^{-2} mbar with a yield of 150 mg (0.7 mmol, 35%).

mp.: −26 °C.

^{1}H NMR [600 MHz, C_6D_6] δ (ppm) 0.93 (s, 6H, N-$C(CH_3)_2$), 1.04 (s, 9H, *t*Bu), 1.05 (s, 6H, N-$C(CH_3)_2$), 1.35--1.37 (m, 3H, CH_2), 1.43--1.48 (m, 2H, CH_2), 1.58--1.66 (m, 1H, CH_2), 2.39 (s, 2H, N-CH_2).

^{13}C NMR [151 MHz, C_6D_6] δ (ppm) 18.3 (t, CH_2), 21.4 (q, 2/6-Me), 30.9 (s, *t*Bu), 31.4 (q, *t*Bu), 36.3 (q, 2/6-Me), 41.9 (t, CH_2), 54.7 (s, N-C), 56.0 (t, N-CH_2).

HRMS: $C_{14}H_{30}N$: $[M + H]^+$ calc.: 212.2373, exp.: 212.2375.

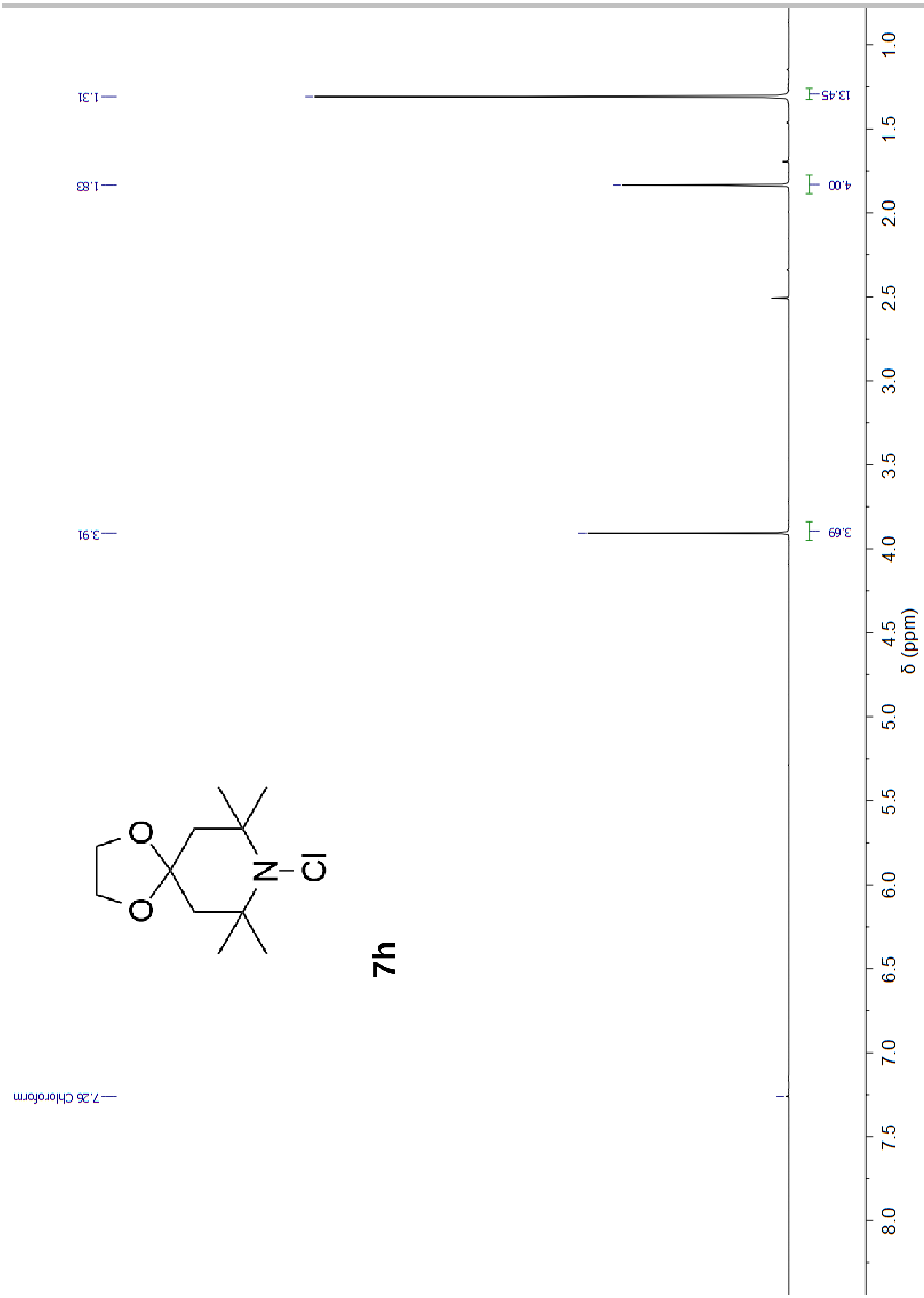
1.31
1.83
3.91
7.26 Chloroform
13.45
4.00
3.69
N
Cl
O
O
7h
δ (ppm)
1.0
1.5
2.0
2.5
3.0
3.5
4.0
4.5
5.0
5.5
6.0
6.5
7.0
7.5
8.0

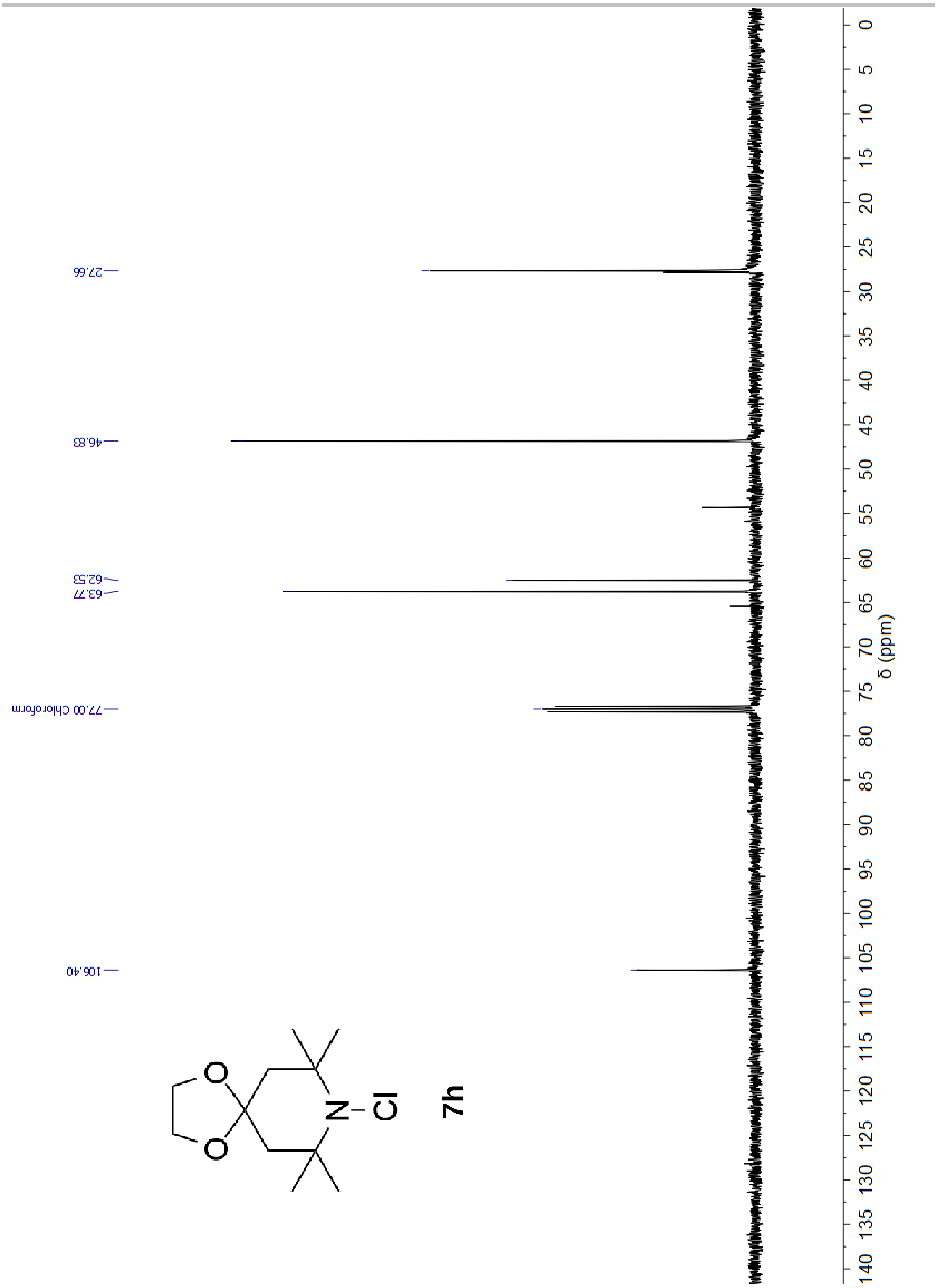
27.66
46.83
62.53
63.77
77.00 chloroform
106.40
7h
δ (ppm)

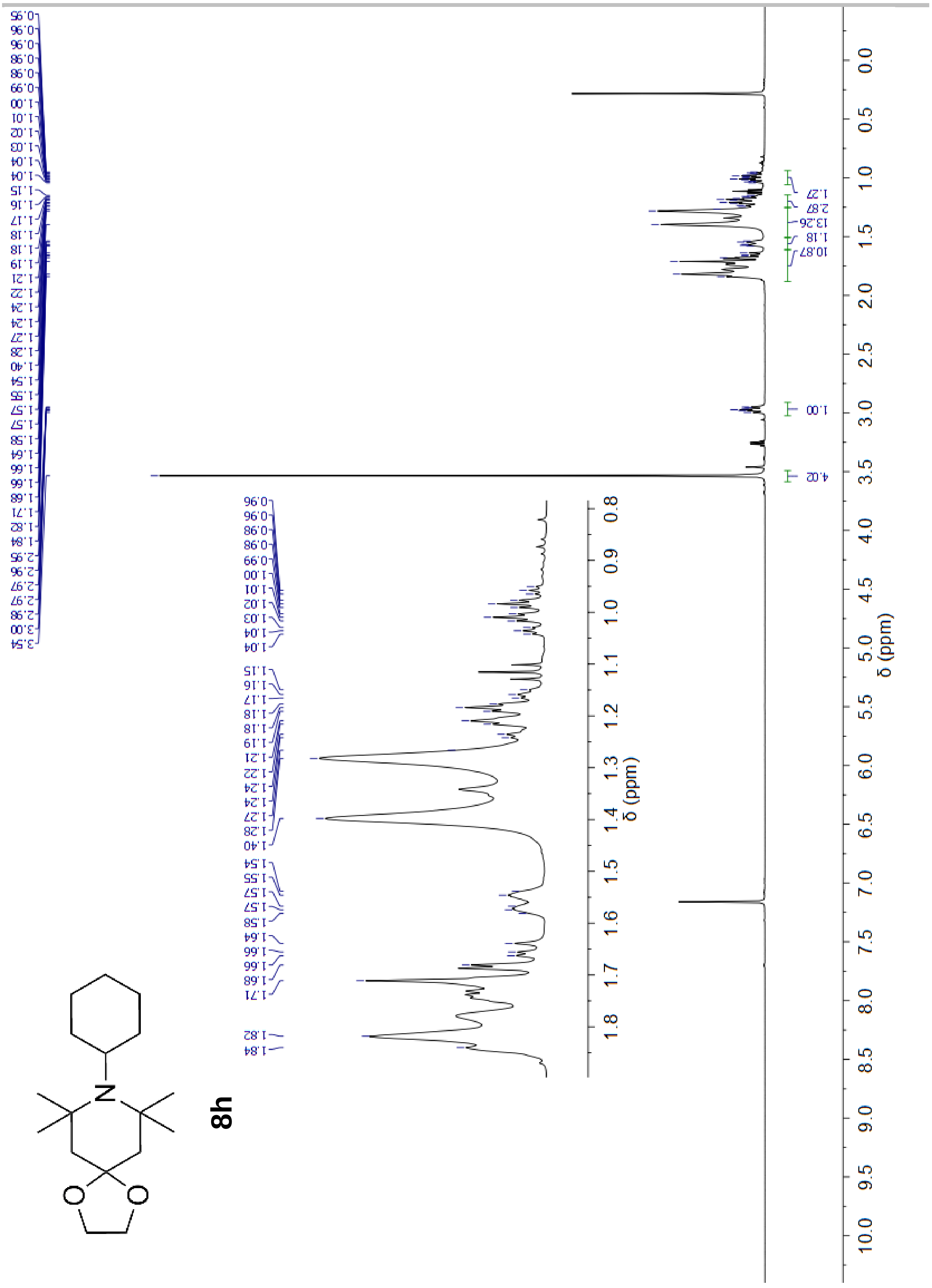
8h
δ (ppm)

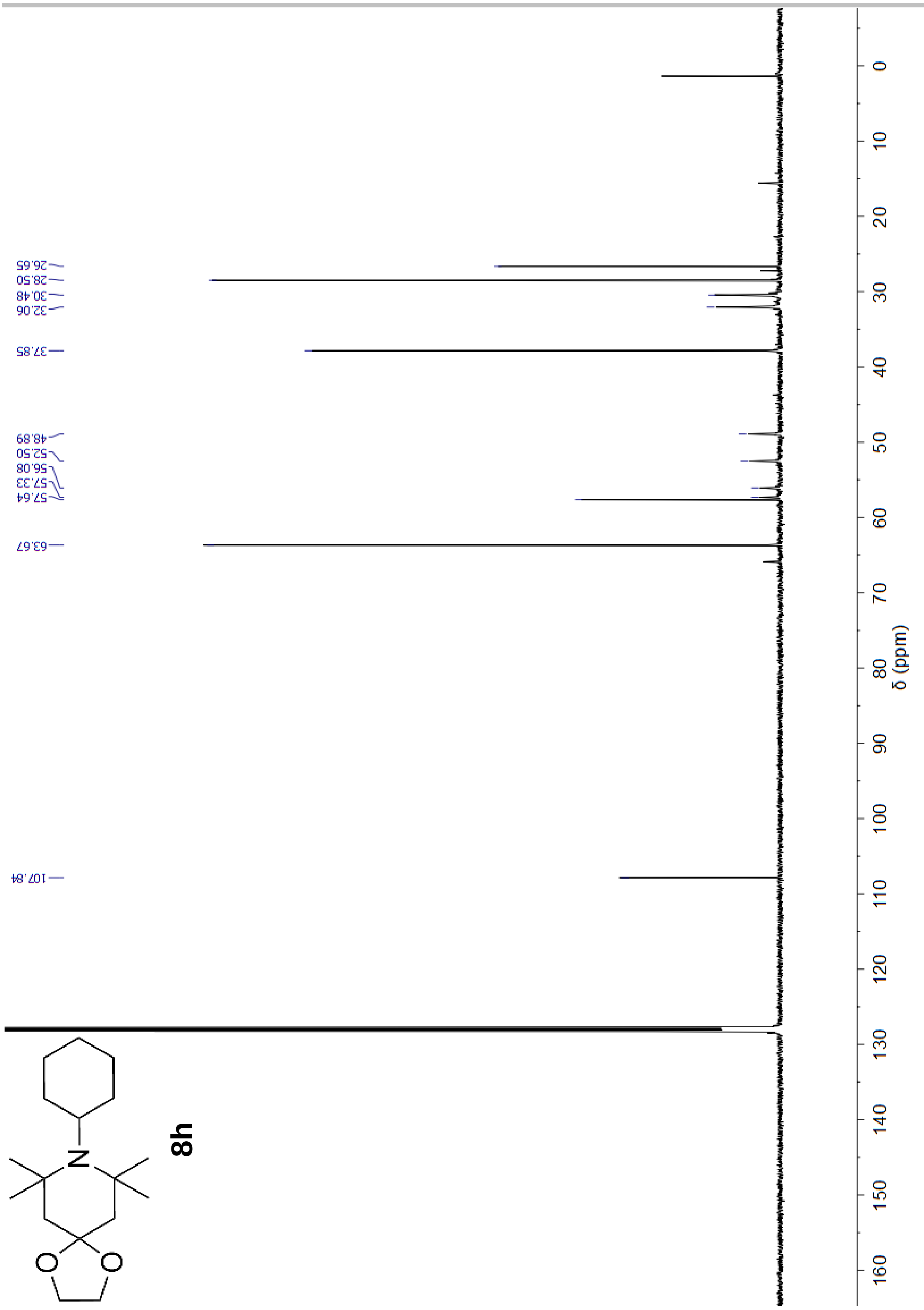
26.65
28.50
30.48
32.06
37.85
48.89
52.50
56.08
57.33
57.64
63.67
107.84
δ (ppm)
8h

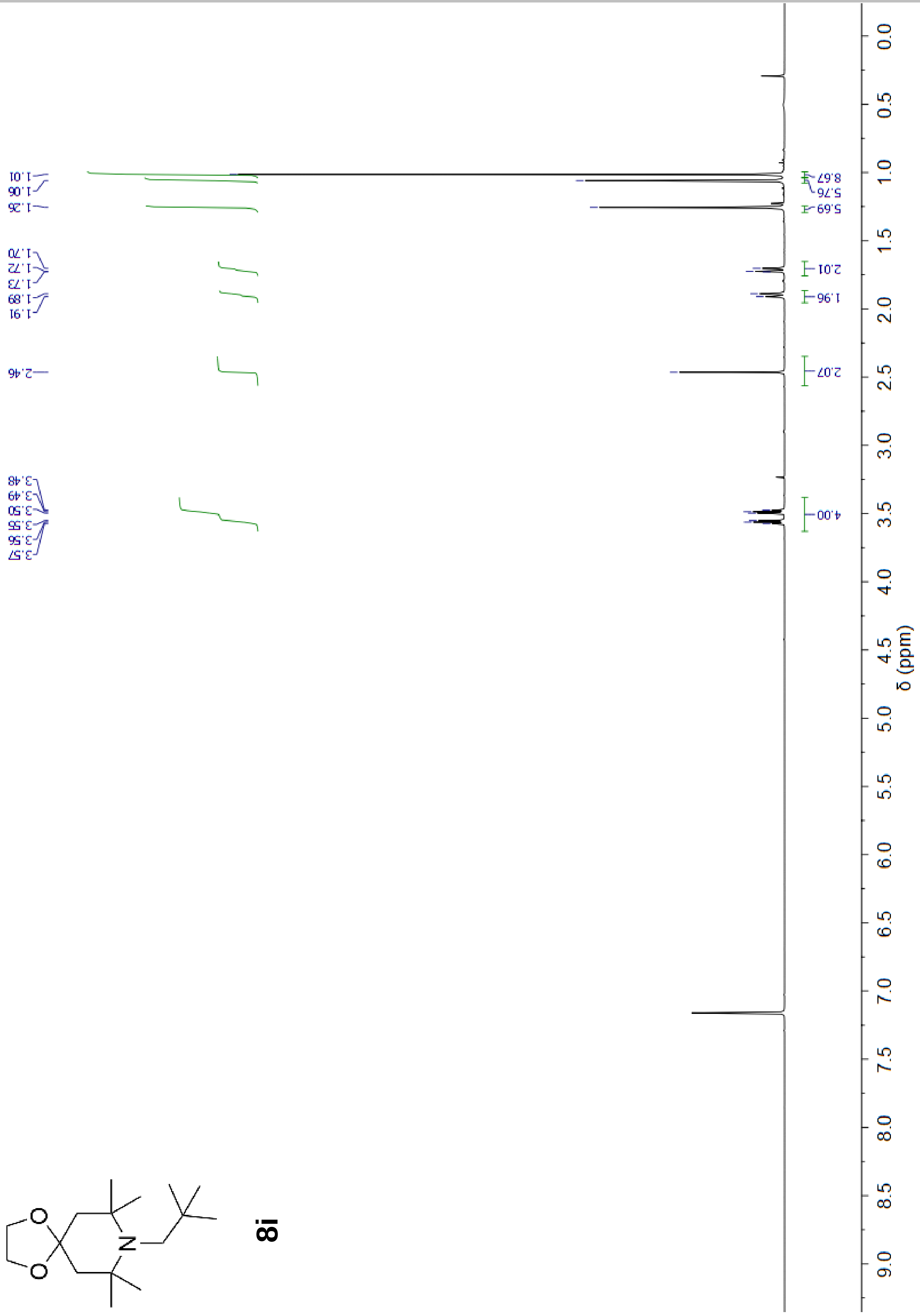
1.01
1.06
1.26
1.70
1.72
1.73
1.89
1.91
2.46
3.48
3.49
3.50
3.55
3.56
3.57
8.67
5.76
5.69
2.01
1.96
2.07
4.00
0.0
0.5
1.0
1.5
2.0
2.5
3.0
3.5
4.0
4.5
5.0
5.5
6.0
6.5
7.0
7.5
8.0
8.5
9.0
δ (ppm)
O
O
N

8i

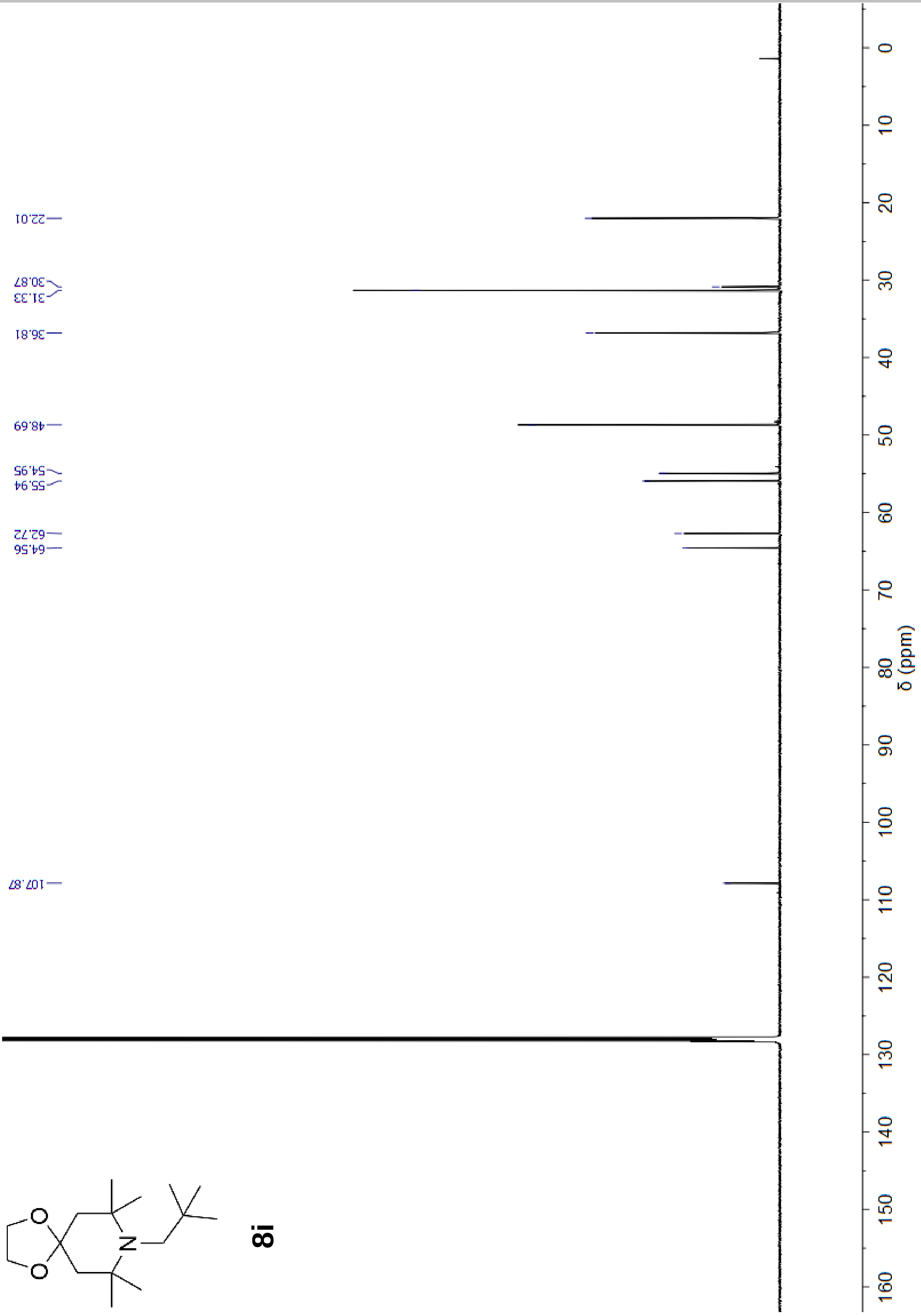
22.01
30.87
31.33
36.81
48.69
54.95
55.94
62.72
64.56
107.87
8i
δ (ppm)

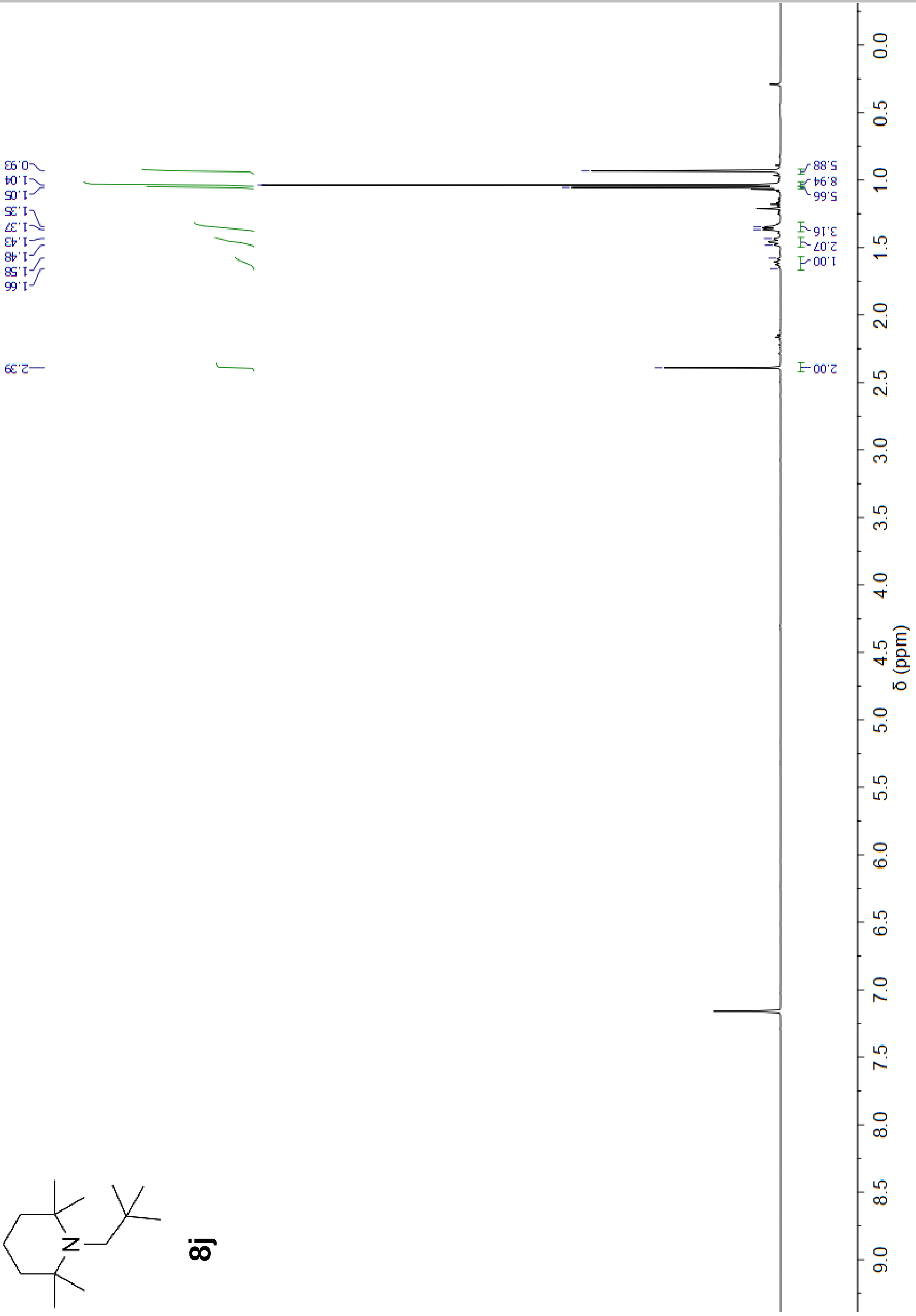
0.93
1.04
1.05
1.35
1.37
1.43
1.48
1.58
1.66
2.39
5.88
8.94
5.66
3.16
2.07
1.00
2.00
N
8j
δ (ppm)

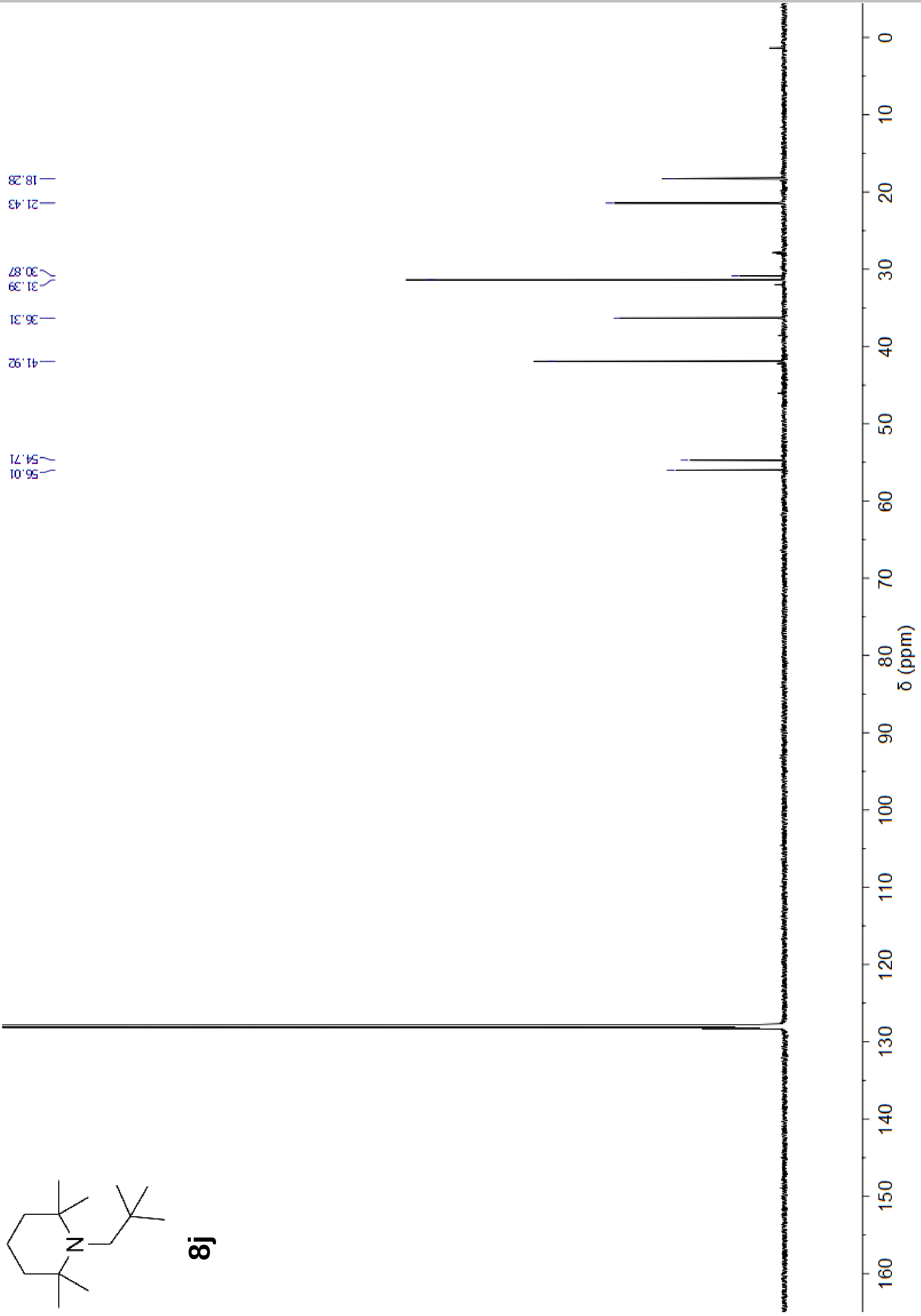

8j

Crystal Structures

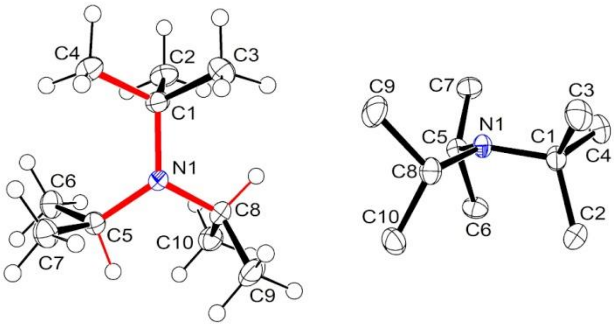

Molecular structure of **8a**.

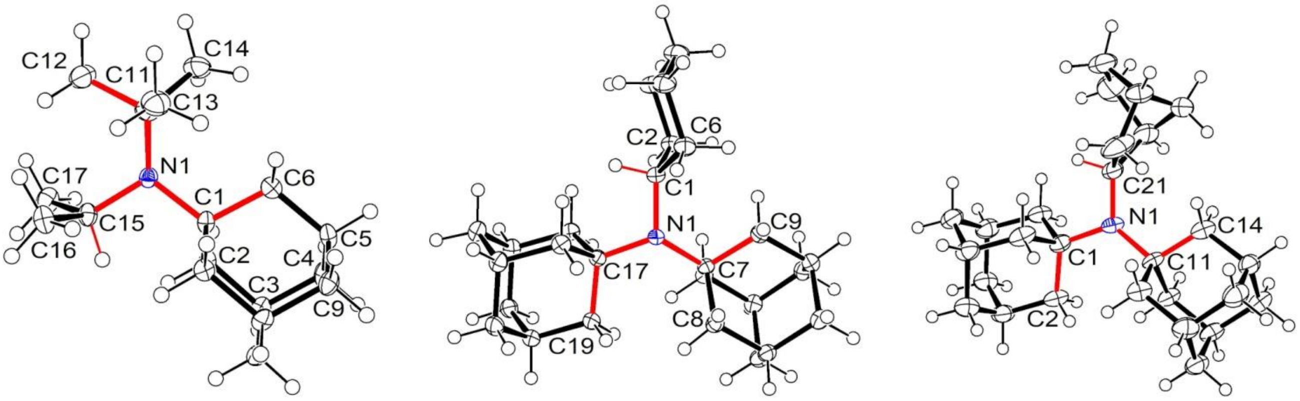

Molecular structures of **8e**, **8f** and **8g**.

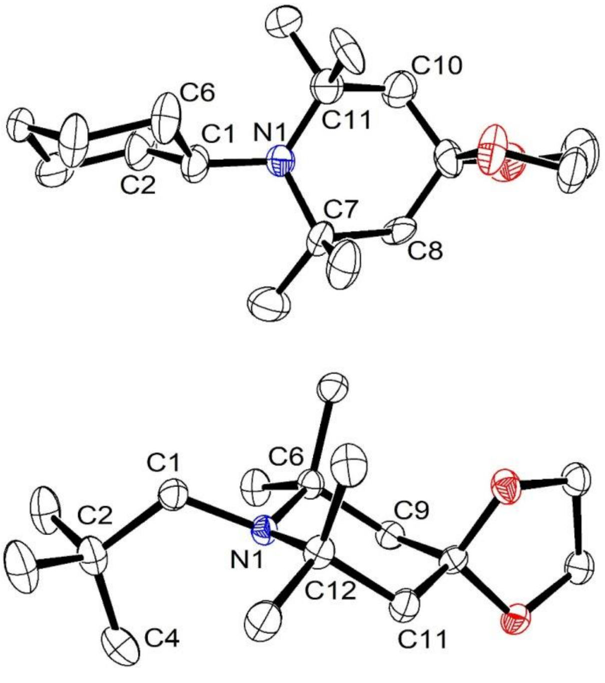

Molecular structures of **8h** and **8i**.

Details of the Single Crystal Structure Determination of Compound 8a (*tert*-butyl-diisopropylamine, CCDC 2002567)

The compound forms a plastic phase upon cooling below 230 K. Below about 205 K this phase becomes thermodynamically unstable and is supercooled. It transforms into an ordered, polycrystalline state between 190 and 180 K. From this polycrystalline phase a single crystal in a 0.3 mm Mark-tube could be grown by slowly heating and tempering around the phase transition point at about 203–205 K. The data set was collected on a Bruker SMART. Using Olex2 [S-1], the structure was solved with the Superflip [S-4] structure solution program using Charge Flipping and refined with ShelXL [S-5] using Least Squares minimisation.

The structure determination of a measurement at 200 K revealed a disordered structure in the space group $P2_1/m$. When cooling to 100 K further ordering takes place and the compound can be described in the space group $P2_1/c$. Weak residual electron density was observed in the difference Fourier map, corresponding to about 3% of *tert*-butyl group situated on the isopropyl group position. This type of disorder is observed for the crystals measured at 200 K, too. Because of the low occupancy, the corresponding residual electron density was neglected in the subsequent refinement. All hydrogen atoms were located in the difference Fourier map and their positions as well as displacement parameters were refined freely. The distance of the nitrogen atom N1 from the plane defined by the neighboring carbon atoms C1-C8-C5 is 0.2537(3) Å.

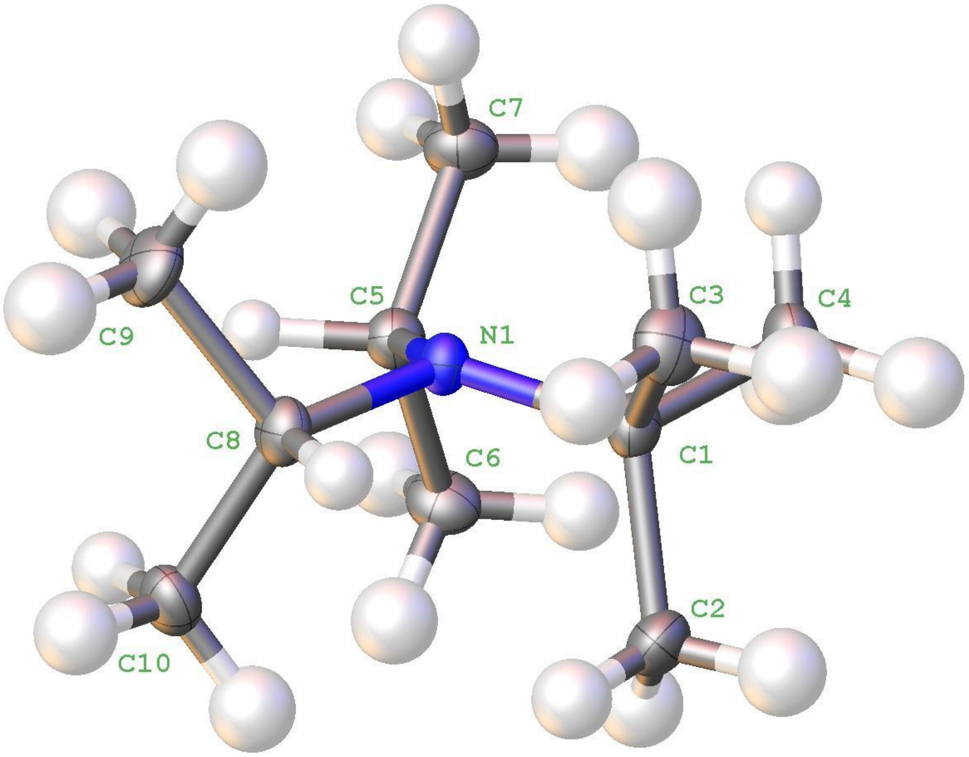

Figure S-1: View of *tert*-butyl-diisopropylamine (8a). Carbon atoms represented as grey and nitrogen atoms as blue 50 % probability ellipsoids.

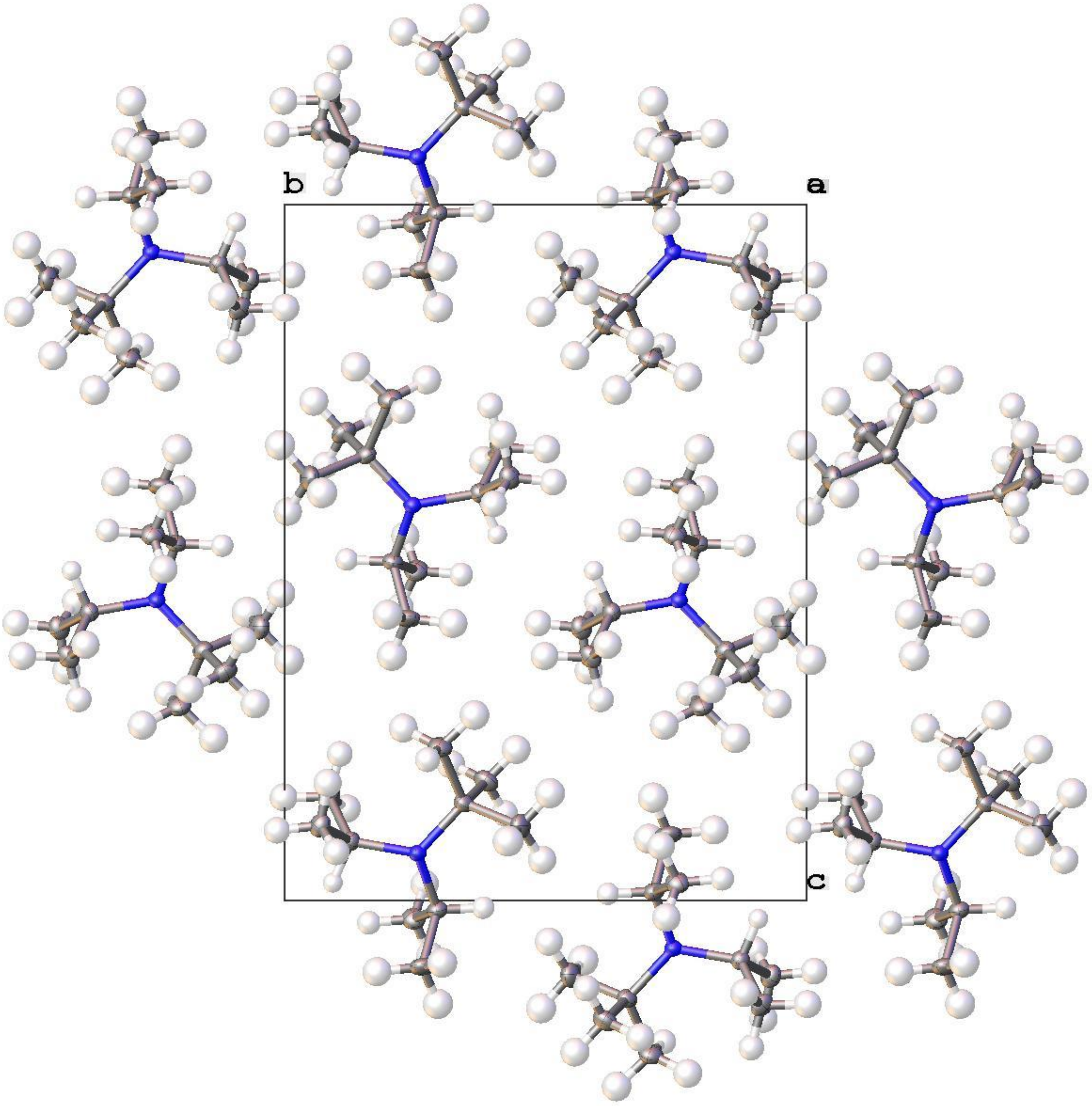

Figure S-2: View along the crystallographic a direction of the packing of *tert*-butyl-diisopropylamine. Carbon atoms represented as grey and nitrogen atoms as blue 50 % probability ellipsoids.

Table S-1 Crystal data and structure refinement for **8a** (CCDC 2002567).

Identification code	amin09o06
Empirical formula	$C_{10}H_{23}N$
Formula weight	157.29
Temperature/K	100
Crystal system	monoclinic
Space group	$P2_1/n$
a/Å	6.3045(10)
b/Å	11.2957(17)
c/Å	15.212(2)
α/°	90
β/°	97.947(2)
γ/°	90
Volume/Å^3	1072.9(3)
Z	4
ρ_{calc}g/cm^3	0.974
μ/mm^{-1}	0.056
F(000)	360.0
Crystal size/mm^3	0.6 × 0.3 × 0.3
Radiation	MoKα (λ = 0.71073)
2Θ range for data collection/°	4.506 to 62.936
Index ranges	-8 ≤ h ≤ 8, -16 ≤ k ≤ 15, -21 ≤ l ≤ 22
Reflections collected	12400
Independent reflections	3276 [R_{int} = 0.0248, R_{sigma} = 0.0215]
Data/restraints/parameters	3276/0/192
Goodness-of-fit on F^2	1.072
Final R indexes [I>=2σ (I)]	R_1 = 0.0539, wR_2 = 0.1395
Final R indexes [all data]	R_1 = 0.0594, wR_2 = 0.1447
Largest diff. peak/hole / e Å^{-3}	0.56/-0.20

Table S-2 Fractional Atomic Coordinates (×10⁴) and Equivalent Isotropic Displacement Parameters (Å²×10³) for amine **8a**. U_{eq} is defined as 1/3 of of the trace of the orthogonalised U_{IJ} tensor.

Atom	*x*	*y*	*z*	U(eq)
N1	3087.5(11)	2450.6(6)	5683.4(4)	16.90(17)
C1	3245.1(13)	1562.5(7)	6403.6(5)	20.71(19)
C2	5550.2(15)	1139.6(9)	6719.2(6)	26.9(2)
C3	1873.6(17)	480.5(8)	6069.0(7)	28.9(2)
C4	2253.4(16)	2018.9(9)	7205.4(6)	27.7(2)
C5	3321.2(13)	3733.4(7)	5853.6(5)	19.35(18)
C6	5281.9(14)	4145.9(8)	6498.6(6)	24.5(2)
C7	1274.9(14)	4316.0(8)	6081.6(6)	24.6(2)
C8	3914.7(14)	2073.1(8)	4867.7(5)	21.62(19)
C9	2288.9(17)	2357.1(10)	4051.8(6)	30.2(2)
C10	6148.6(16)	2526.5(8)	4740.4(6)	25.9(2)

Table S-3 Bond Lengths for **8a**.

Atom	Atom	Length/Å	Atom	Atom	Length/Å
N1	C1	1.4786(10)	C1	C4	1.5346(12)
N1	C5	1.4757(10)	C5	C6	1.5403(12)
N1	C8	1.4739(10)	C5	C7	1.5302(12)
C1	C2	1.5420(12)	C8	C9	1.5304(13)
C1	C3	1.5423(12)	C8	C10	1.5358(13)

Table S-4 Bond Angles for **8a**.

Atom	Atom	Atom	Angle/˚	Atom	Atom	Atom	Angle/˚
C5	N1	C1	122.77(6)	C4	C1	C3	104.90(7)
C8	N1	C1	115.60(6)	N1	C5	C6	117.43(7)
C8	N1	C5	113.17(6)	N1	C5	C7	113.28(7)
N1	C1	C2	113.87(7)	C7	C5	C6	110.67(7)
N1	C1	C3	108.42(7)	N1	C8	C9	110.30(7)
N1	C1	C4	111.59(7)	N1	C8	C10	116.56(7)
C2	C1	C3	108.57(7)	C9	C8	C10	110.54(8)
C4	C1	C2	109.06(7)				

Details of the Single Crystal Structure Determination of Compound 8e (1-adamantyl-*tert*-butylisopropylamine, CCDC 2002569)

A suitable crystal was measured on a Bruker SMART Platform diffractometer, equipped with an Apex I-detector. Using Olex2 [S-1], the structure was solved with the ShelXS [S-2] structure solution program using Direct Methods and refined with the ShelXL [S-5] refinement package using Least Squares minimization. The weakly diffracting crystals turned out to be twinned (monoclinic twin with β close to 90° and a volume ratio of almost 1:1, emulating the orthorhombic Laue class mmm, twin matrix -1 0 0 0 -1 0 0 0 1). The asymmetric unit contains two crystallographically independent molecules, with practically identical conformation. All hydrogen atom positions can be located in the difference Fourier map. However, a free refinement of the displacement parameters was not possible. Therefore, the hydrogen atoms were placed at calculated positions and refined according to the riding model. The distance of the nitrogen atoms N1 and N1A from the plane defined by C11-C1-C15 and C11A-C1A-C15A is 0.2588(19) Å and 0.2585(19) Å, respectively.

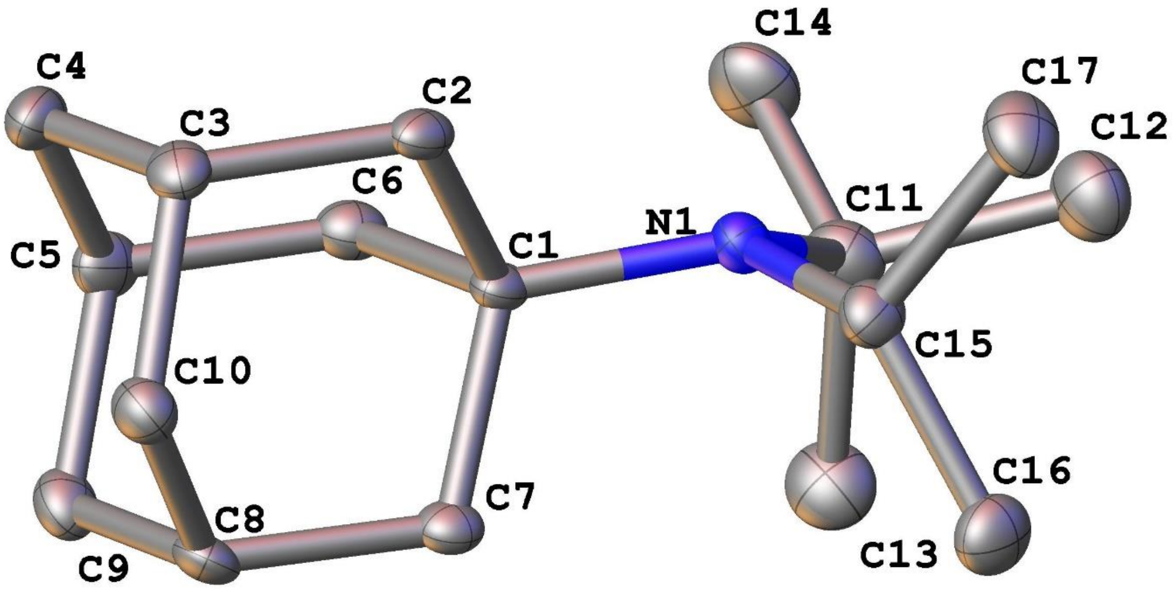

Figure S-3: View of one of the molecules in the crystal structure of 1-adamantyl-*tert*-butylisopropylamine (compound 8e). The hydrogen atoms were omitted for clarity. Carbon atoms represented as grey and nitrogen atoms as blue 50 % probability ellipsoids.

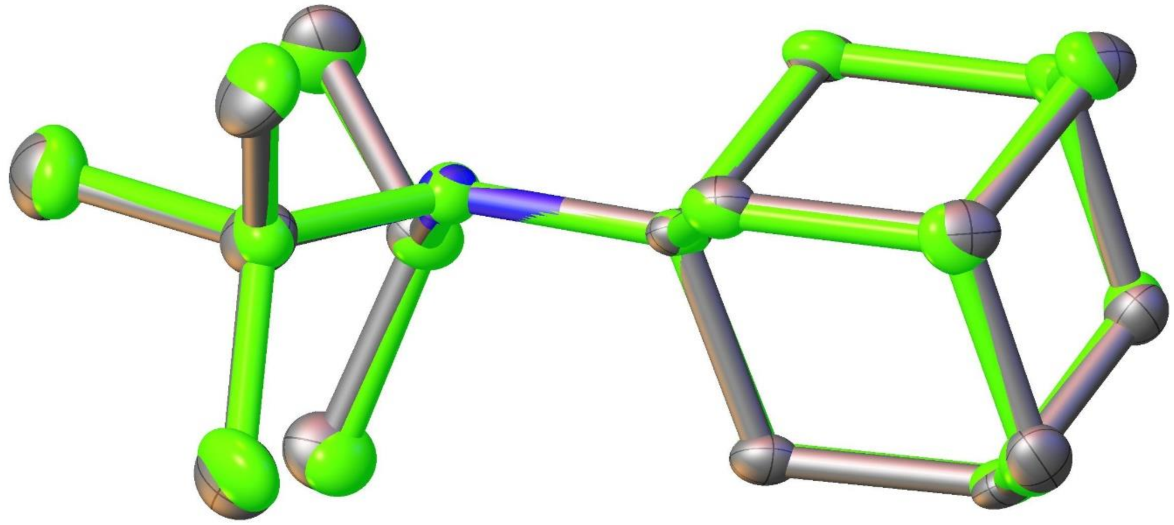

Figure S-4: An overlay of the two crystallographically independent molecules in the crystal structure of 1-adamantyl-*tert*-butylisopropylamin. Carbon atoms represented as grey and nitrogen atoms as blue 50 % probability ellipsoids. The second crystallographically independent molecule is shown in green.

Table S-5 Crystal data and structure refinement for compound **8e** (CCDC 2002569)

Identification code	amin10p06
Empirical formula	$C_{17}H_{31}N$
Formula weight	249.43
Temperature/K	100.0
Crystal system	monoclinic
Space group	$P2_1/c$
a/Å	18.776(5)
b/Å	6.4752(18)
c/Å	24.315(6)
α/°	90
β/°	90.017(4)
γ/°	90
Volume/Å^3	2956.2(14)
Z	8
ρ_{calc}g/cm^3	1.121
μ/mm^{-1}	0.063
F(000)	1120.0
Crystal size/mm^3	0.711 × 0.216 × 0.086
Radiation	MoKα (λ = 0.71073)
2Θ range for data collection/°	1.674 to 50.816
Index ranges	-22 ≤ h ≤ 22, -7 ≤ k ≤ 7, -29 ≤ l ≤ 29
Reflections collected	33220
Independent reflections	5434 [R_{int} = 0.0607, R_{sigma} = 0.0371]
Data/restraints/parameters	5434/0/336
Goodness-of-fit on F^2	1.034
Final R indexes [I>=2σ (I)]	R_1 = 0.0435, wR_2 = 0.0987
Final R indexes [all data]	R_1 = 0.0556, wR_2 = 0.1044
Largest diff. peak/hole / e Å^{-3}	0.26/-0.19

Table S-6 Fractional Atomic Coordinates (×10^4) and Equivalent Isotropic Displacement Parameters (Å^2×10^3) for amine **8e**. U_{eq} is defined as 1/3 of of the trace of the orthogonalised U_{IJ} tensor.

Atom	*x*	*y*	*z*	U(eq)
N1	1694.7(9)	7134(2)	4472.8(7)	16.4(4)
C1	1177(1)	7742(3)	4022.6(9)	15.0(4)
C2	1129.8(11)	5927(3)	3610.9(8)	16.0(4)
C3	636.2(11)	6407(3)	3122.4(9)	17.5(5)
C4	-116.1(11)	6768(3)	3326.5(9)	21.3(5)
C5	-100.7(11)	8554(3)	3735.3(9)	19.6(5)
C6	409.6(11)	8070(3)	4218.0(9)	18.3(5)
C7	1395.6(10)	9730(3)	3713.5(8)	16.3(4)
C8	889.9(11)	10176(3)	3230.5(8)	18.1(4)
C9	139.5(11)	10517(3)	3441.9(9)	20.8(5)
C10	897.0(12)	8360(3)	2831.4(8)	19.3(5)
C11	1611.0(13)	7808(3)	5055.6(9)	23.4(5)
C12	2209.4(13)	6878(4)	5415.5(10)	29.5(6)
C13	1625.3(13)	10171(3)	5142.1(9)	29.3(5)
C14	935.0(13)	6934(4)	5320.4(10)	30.1(6)
C15	2426.9(11)	6968(3)	4251.2(9)	19.0(5)
C16	2948.8(11)	8762(3)	4341.2(10)	25.4(5)
C17	2784.9(12)	4891(3)	4370.8(10)	26.9(5)
N1A	6698.0(9)	8117(2)	3020.9(7)	16.7(4)
C1A	6167.3(11)	7807(3)	3473.0(9)	14.0(4)
C2A	6161.8(11)	9811(3)	3821.3(8)	16.3(4)
C3A	5652.2(11)	9650(3)	4306.0(9)	19.0(5)
C4A	4896.1(11)	9323(3)	4094.7(9)	20.6(5)
C5A	4875.9(11)	7350(3)	3751.4(9)	18.3(4)
C6A	5395.5(10)	7526(3)	3271.7(9)	16.0(4)
C7A	6343.9(10)	5965(3)	3854.7(8)	15.7(4)
C8A	5830.2(11)	5835(3)	4337.1(9)	19.3(5)
C9A	5076.9(12)	5518(3)	4117.2(9)	21.4(5)
C10A	5871.0(12)	7825(3)	4670.8(9)	19.0(5)
C11A	6588.5(12)	7248(3)	2456.5(9)	22.1(5)
C12A	7209.5(13)	7884(4)	2090.3(10)	30.7(6)
C13A	6524.4(13)	4883(3)	2431.5(9)	29.8(5)
C14A	5940.1(12)	8204(3)	2163.3(9)	26.7(5)
C15A	7437.5(11)	8193(3)	3254.7(9)	20.0(5)
C16A	7892.8(12)	6230(3)	3239.3(10)	25.5(5)
C17A	7868.5(12)	10068(3)	3074.1(10)	28.3(5)

Table S-7 Bond Lengths for compound **8e** (CCDC 2002569).

Atom	Atom	Length/Å	Atom	Atom	Length/Å
N1	C1	1.516(3)	N1A	C1A	1.497(3)
N1	C11	1.491(3)	N1A	C11A	1.497(3)
N1	C15	1.481(3)	N1A	C15A	1.501(3)
C1	C2	1.546(3)	C1A	C2A	1.549(3)
C1	C6	1.532(3)	C1A	C6A	1.540(3)
C1	C7	1.546(3)	C1A	C7A	1.547(3)
C2	C3	1.538(3)	C2A	C3A	1.522(3)
C3	C4	1.515(3)	C3A	C4A	1.524(3)
C3	C10	1.529(3)	C3A	C10A	1.534(3)
C4	C5	1.525(3)	C4A	C5A	1.527(3)
C5	C6	1.547(3)	C5A	C6A	1.525(3)
C5	C9	1.526(3)	C5A	C9A	1.530(3)
C7	C8	1.537(3)	C7A	C8A	1.521(3)
C8	C9	1.516(3)	C8A	C9A	1.526(3)
C8	C10	1.525(3)	C8A	C10A	1.524(3)
C11	C12	1.546(3)	C11A	C12A	1.524(3)
C11	C13	1.545(3)	C11A	C13A	1.538(3)
C11	C14	1.532(3)	C11A	C14A	1.540(3)
C15	C16	1.535(3)	C15A	C16A	1.532(3)
C15	C17	1.531(3)	C15A	C17A	1.524(3)

Table S-8 Bond Angles for compound **8e** (CCDC 2002569).

Atom	Atom	Atom	Angle/˚	Atom	Atom	Atom	Angle/˚
C11	N1	C1	122.86(17)	C1A	N1A	C15A	110.00(16)
C15	N1	C1	110.57(15)	C11A	N1A	C1A	122.11(17)
C15	N1	C11	117.71(18)	C11A	N1A	C15A	119.09(17)
N1	C1	C2	107.87(15)	N1A	C1A	C2A	107.09(15)
N1	C1	C6	114.52(17)	N1A	C1A	C6A	114.12(17)
N1	C1	C7	113.39(15)	N1A	C1A	C7A	113.64(15)
C2	C1	C7	109.47(17)	C6A	C1A	C2A	105.44(15)
C6	C1	C2	104.62(16)	C6A	C1A	C7A	107.52(16)
C6	C1	C7	106.55(15)	C7A	C1A	C2A	108.61(17)
C3	C2	C1	112.37(15)	C3A	C2A	C1A	111.74(16)
C4	C3	C2	109.87(17)	C2A	C3A	C4A	109.53(17)
C4	C3	C10	108.83(16)	C2A	C3A	C10A	109.40(16)
C10	C3	C2	109.37(16)	C4A	C3A	C10A	109.70(17)

C3	C4	C5	108.22(16)	C3A	C4A	C5A	108.87(16)
C4	C5	C6	110.64(17)	C4A	C5A	C9A	108.96(18)
C4	C5	C9	109.43(18)	C6A	C5A	C4A	109.84(16)
C9	C5	C6	109.89(16)	C6A	C5A	C9A	110.18(16)
C1	C6	C5	112.04(17)	C5A	C6A	C1A	111.59(17)
C8	C7	C1	111.33(16)	C8A	C7A	C1A	111.67(16)
C9	C8	C7	109.99(17)	C7A	C8A	C9A	108.98(17)
C9	C8	C10	109.65(17)	C7A	C8A	C10A	109.39(16)
C10	C8	C7	109.62(16)	C10A	C8A	C9A	110.29(17)
C8	C9	C5	108.19(16)	C8A	C9A	C5A	109.14(16)
C8	C10	C3	109.90(17)	C8A	C10A	C3A	109.26(18)
N1	C11	C12	110.31(18)	N1A	C11A	C12A	109.22(18)
N1	C11	C13	114.67(17)	N1A	C11A	C13A	114.90(18)
N1	C11	C14	112.25(19)	N1A	C11A	C14A	112.43(17)
C13	C11	C12	107.23(19)	C12A	C11A	C13A	107.81(19)
C14	C11	C12	102.74(18)	C12A	C11A	C14A	103.05(18)
C14	C11	C13	108.85(19)	C13A	C11A	C14A	108.67(19)
N1	C15	C16	119.07(17)	N1A	C15A	C16A	118.67(17)
N1	C15	C17	113.72(17)	N1A	C15A	C17A	114.10(17)
C17	C15	C16	110.93(18)	C17A	C15A	C16A	110.94(18)

Details of the Single Crystal Structure Determination of Compound 8f (di-(1-adamantyl)cyclohexylamine, CCDC 2002571)

Fiber-shaped crystals were grown from a dibutylether/ethanol mixture and measured at 100 K on a Rigaku-Oxford XtaLAB Synergy Dualflex diffractometer equipped with a Pilatus 300K detector. Using Olex2 [S-1], the structure was solved with the ShelXT [S-3] structure solution program using Intrinsic Phasing and refined with the ShelXL [S-5] refinement package using Least Squares minimization. All hydrogen atoms were found in the difference Fourier map and freely refined. The distance of the nitrogen atom from the plane defined by C1-C7-C17 is 0.2410(18) Å.

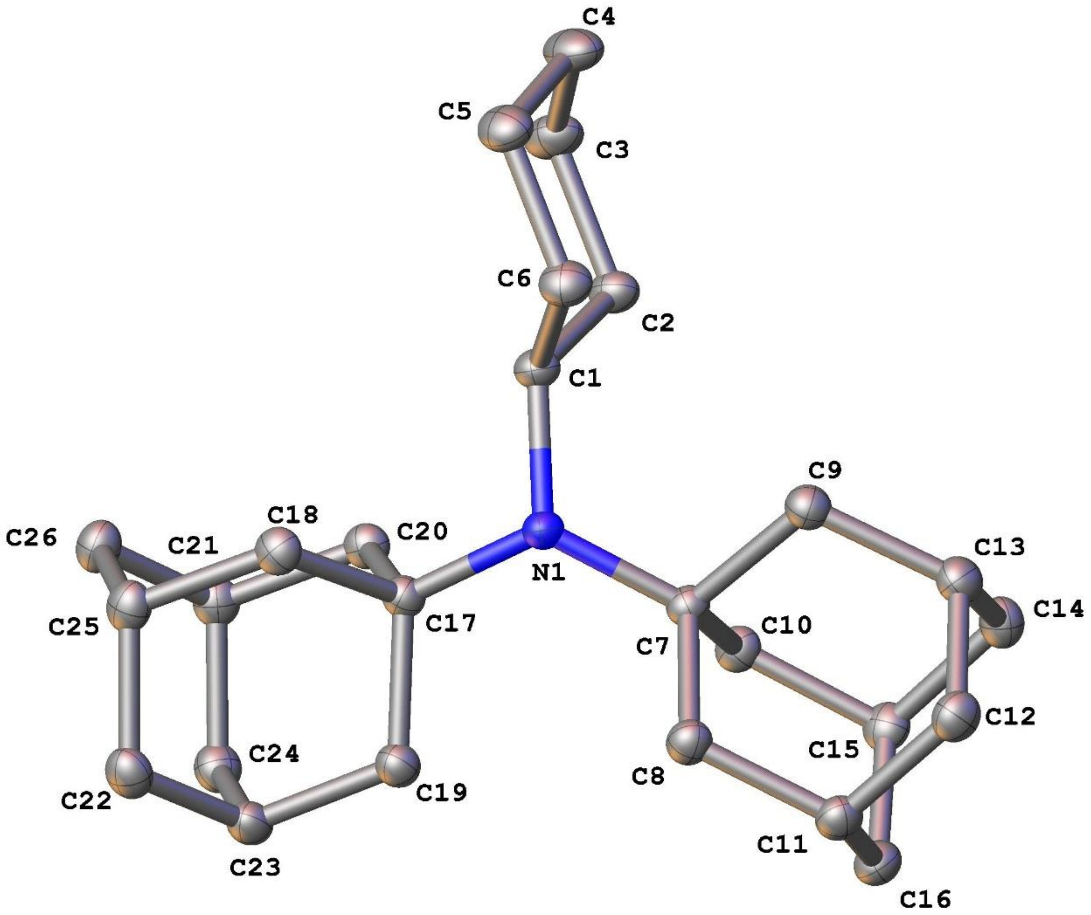

Figure S-5: View of di-(1-adamantyl)cyclohexylamine (8f). The hydrogen atoms were omitted for clarity. Carbon atoms represented as grey and nitrogen atoms as blue 50 % probability ellipsoids.

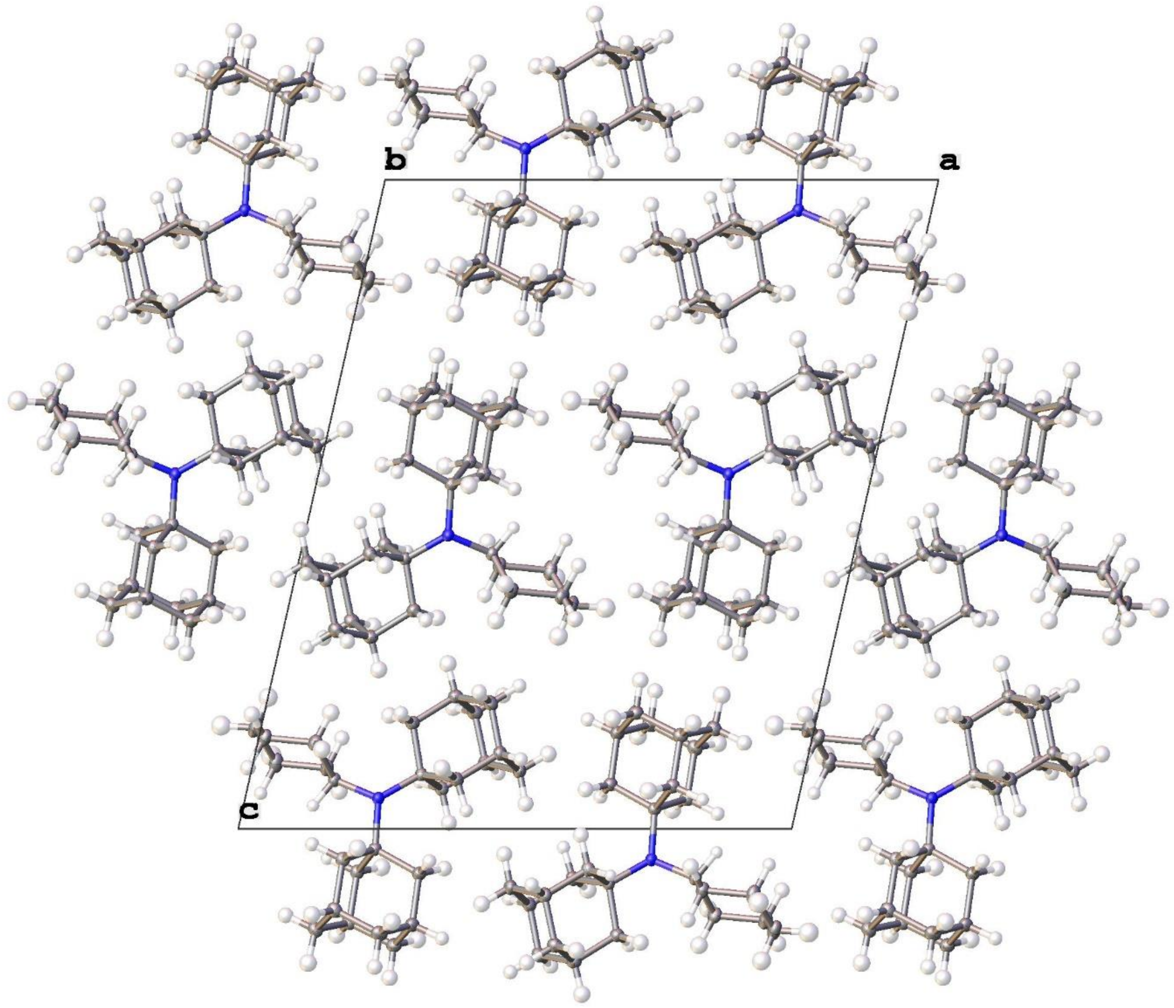

Figure S-6: View along the crystallographic b direction of the packing of di-(1-adamantyl)cyclohexylamine. Carbon atoms represented as grey and nitrogen atoms as blue 50 % probability ellipsoids.

Table S-9 Crystal data and structure refinement for compound **8f** (CCDC 2002571).

Identification code	wit_sol05o10
Empirical formula	$C_{26}H_{41}N$
Formula weight	367.60
Temperature/K	100
Crystal system	monoclinic
Space group	$P2_1/n$
a/Å	16.46750(10)
b/Å	6.46350(10)
c/Å	19.8240(2)
α/°	90
β/°	102.7450(10)
γ/°	90
Volume/Å^3	2058.03(4)
Z	4
ρ_{calc}g/cm^3	1.186
μ/mm^{-1}	0.493
F(000)	816.0
Crystal size/mm^3	0.168 × 0.129 × 0.074
Radiation	CuKα (λ = 1.54184)
2Θ range for data collection/°	6.33 to 160.578
Index ranges	-20 ≤ h ≤ 20, -8 ≤ k ≤ 7, -25 ≤ l ≤ 25
Reflections collected	232051
Independent reflections	4451 [R_{int} = 0.0756, R_{sigma} = 0.0128]
Data/restraints/parameters	4451/0/409
Goodness-of-fit on F^2	1.114
Final R indexes [I>=2σ (I)]	R_1 = 0.0579, wR_2 = 0.1342
Final R indexes [all data]	R_1 = 0.0585, wR_2 = 0.1346
Largest diff. peak/hole / e Å^{-3}	0.38/-0.24

Table S-10 Fractional Atomic Coordinates (×10[4]) and Equivalent Isotropic Displacement Parameters (Å[2]×10[3]) for amine **8f**. U_{eq} is defined as 1/3 of of the trace of the orthogonalised U_{IJ} tensor.

Atom	*x*	*y*	*z*	U(eq)
N1	2591.5(9)	3043(2)	5471.7(7)	17.2(3)
C1	3461.2(10)	2339(3)	5757.4(9)	17.1(4)
C2	3645.6(11)	31(3)	5942.1(10)	20.4(4)
C3	4581.3(12)	-362(3)	6015.6(11)	26.6(4)
C4	5107.9(12)	1050(3)	6558.9(12)	29.3(5)
C5	4899.2(12)	3337(3)	6405.7(11)	25.3(4)
C6	3964.9(11)	3728(3)	6331.8(10)	22.0(4)
C7	1919(1)	2489(3)	5841.8(9)	15.9(3)
C8	1250.1(11)	4224(3)	5761.8(9)	19.2(4)
C9	2278.9(11)	2404(3)	6633.1(9)	19.4(4)
C10	1489.0(11)	392(3)	5621.4(9)	18.4(4)
C11	574.2(11)	3739(3)	6165.5(9)	20.1(4)
C12	951.2(12)	3569(3)	6938.4(10)	22.2(4)
C13	1612.3(11)	1868(3)	7042.0(9)	21.2(4)
C14	1201.8(12)	-202(3)	6794.1(9)	21.6(4)
C15	805.2(11)	-54(3)	6019.8(9)	20.3(4)
C16	155.6(11)	1687(3)	5897(1)	22.0(4)
C17	2442.8(10)	3229(3)	4690.9(9)	16.5(3)
C18	2986.6(11)	5062(3)	4531.4(9)	19.1(4)
C19	1545.9(11)	3814(3)	4312.0(9)	18.9(4)
C20	2673.8(11)	1231(3)	4338.5(9)	18.3(4)
C21	2921.5(12)	5330(3)	3751.2(9)	20.8(4)
C22	2021.4(12)	5825(3)	3390.7(10)	22.4(4)
C23	1463.0(11)	4053(3)	3524.6(9)	20.3(4)
C24	1711.3(11)	2059(3)	3208.3(9)	21.1(4)
C25	2609.3(11)	1542(3)	3558.1(9)	20.0(4)
C26	3193.1(12)	3318(3)	3452.1(10)	21.6(4)

Table S-11 Bond Lengths for compound **8f** (CCDC 2002571).

Atom	Atom	Length/Å	Atom	Atom	Length/Å
N1	C1	1.490(2)	C12	C13	1.529(3)
N1	C7	1.500(2)	C13	C14	1.530(3)
N1	C17	1.518(2)	C14	C15	1.533(2)
C1	C2	1.550(2)	C15	C16	1.534(3)
C1	C6	1.541(2)	C17	C18	1.559(2)
C2	C3	1.537(2)	C17	C19	1.549(2)
C3	C4	1.527(3)	C17	C20	1.555(2)
C4	C5	1.533(3)	C18	C21	1.536(2)
C5	C6	1.534(3)	C19	C23	1.544(2)
C7	C8	1.555(2)	C20	C25	1.540(2)
C7	C9	1.551(2)	C21	C22	1.531(3)
C7	C10	1.547(2)	C21	C26	1.536(3)
C8	C11	1.540(2)	C22	C23	1.528(3)
C9	C13	1.541(2)	C23	C24	1.528(3)
C10	C15	1.539(2)	C24	C25	1.525(3)
C11	C12	1.525(3)	C25	C26	1.541(3)
C11	C16	1.535(3)			

Table S-12 Bond Angles for compound **8f** (CCDC 2002571).

Atom	Atom	Atom	Angle/˚	Atom	Atom	Atom	Angle/˚
C1	N1	C7	119.49(13)	C13	C14	C15	109.10(15)
C1	N1	C17	109.70(13)	C14	C15	C10	109.10(15)
C7	N1	C17	123.17(13)	C14	C15	C16	109.45(15)
N1	C1	C2	120.37(14)	C16	C15	C10	110.18(15)
N1	C1	C6	114.72(14)	C15	C16	C11	109.54(15)
C6	C1	C2	109.93(14)	N1	C17	C18	107.24(13)
C3	C2	C1	108.75(15)	N1	C17	C19	115.85(13)
C4	C3	C2	111.70(17)	N1	C17	C20	113.07(14)
C3	C4	C5	111.65(16)	C19	C17	C18	104.21(14)
C4	C5	C6	110.74(16)	C19	C17	C20	106.64(14)
C5	C6	C1	109.85(16)	C20	C17	C18	109.36(14)
N1	C7	C8	111.15(14)	C21	C18	C17	111.97(14)
N1	C7	C9	110.15(13)	C23	C19	C17	112.14(14)
N1	C7	C10	114.34(14)	C25	C20	C17	111.92(14)
C9	C7	C8	103.47(14)	C22	C21	C18	109.81(15)
C10	C7	C8	109.46(14)	C22	C21	C26	109.35(15)

C10	C7	C9	107.66(14)	C26	C21	C18	109.38(15)
C11	C8	C7	112.17(15)	C23	C22	C21	108.66(15)
C13	C9	C7	112.49(14)	C22	C23	C19	109.15(15)
C15	C10	C7	111.12(14)	C24	C23	C19	111.01(15)
C12	C11	C8	110.86(15)	C24	C23	C22	109.59(15)
C12	C11	C16	109.79(15)	C25	C24	C23	108.57(15)
C16	C11	C8	108.45(15)	C20	C25	C26	109.10(15)
C11	C12	C13	107.85(15)	C24	C25	C20	109.29(14)
C12	C13	C9	109.65(15)	C24	C25	C26	110.25(15)
C12	C13	C14	109.56(15)	C21	C26	C25	109.53(15)
C14	C13	C9	110.07(15)				

Details of the Single Crystal Structure Determination of Compound 8g (di-(1-adamantyl)*exo*-2-norbornylamine, CCDC 2002570)

A suitable crystal was selected and measured at 100 K on a Rigaku-Oxford XtaLAB Synergy Dualflex diffractometer equipped with a Pilatus 300K detector. Using Olex2 [S-1], the structure was solved with the SHELXT [S-3] structure solution program using Intrinsic Phasing and refined with the SHELXL [S-5] refinement package using Least Squares minimisation. The crystal under investigation was twinned (180° rotation around the crystallographic a-axis). The volume ratio of the two individuals is about 0.66:0.33. On the norbornyl site there is residual electron density which can be described by a 10 percent overlay with an adamantyl group. Accordingly, on the adamantyl-positions there is also an overlay with the norbornyl group. Since the data set is already biased due to twinning and the occupancies of the minority orientations are low, we decided not to refine the disorder of the amine as a whole, which would need three independent positions of the molecule. The N-atom is situated 0.243(5) Å above the plane through the atoms C1-C11-C21.The distance C1 – C21 is 2.460(7) Å, C1 – C11 is 2.616(7) and C11 – C21 is 2.595(7) Å.

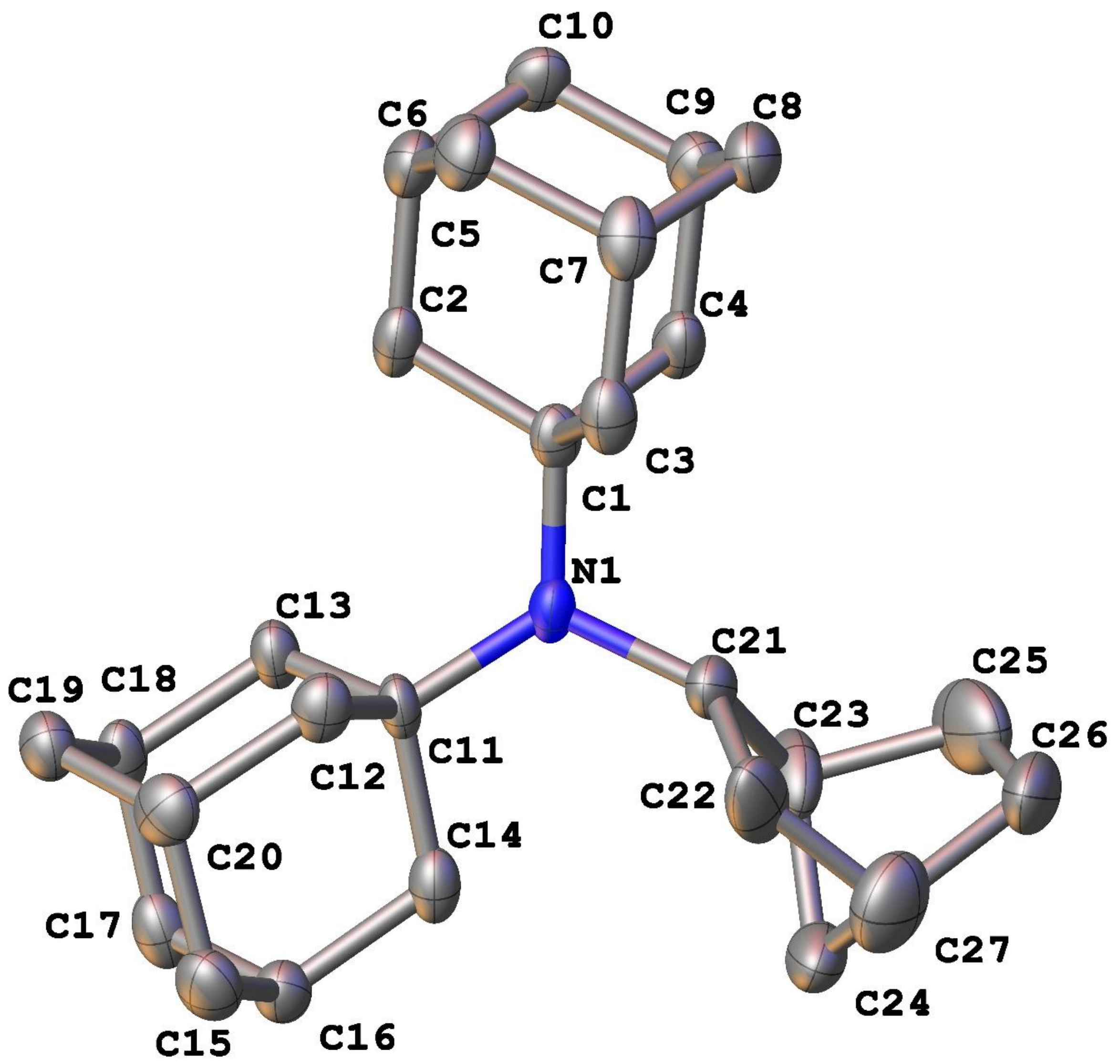

Figure S-7: View of di-(1-adamantyl)*exo*-2-norbornylamine (8g). The hydrogen atoms were omitted for clarity.

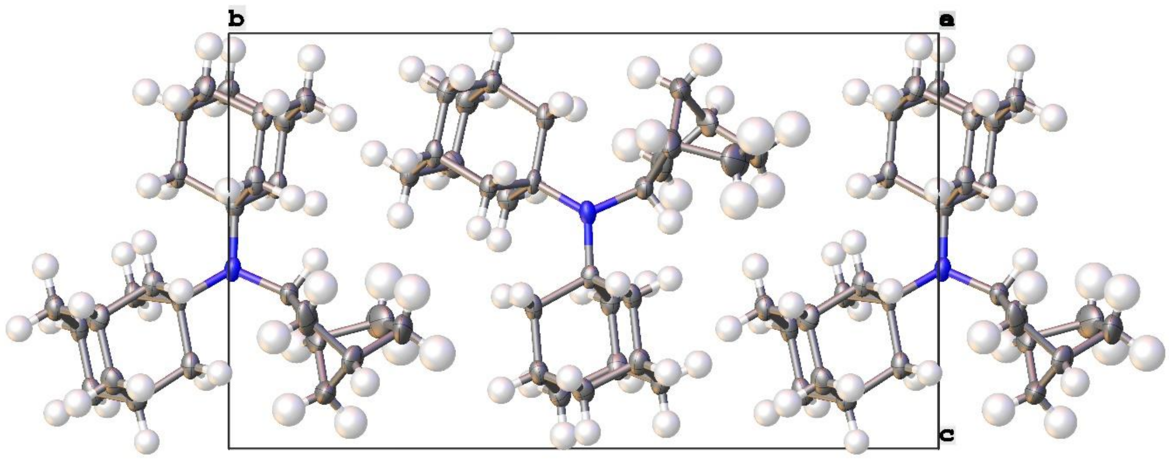

Figure S-8: View along the crystallographic a direction of the packing of di-(1-adamantyl)*exo*-2-norbornylamine.

Table S-13 Crystal data and structure refinement for compound **8g** (CCDC 2002570).

Identification code	sol06o10_1_1_twin1_hklf4
Empirical formula	$C_{27}H_{41}N$
Formula weight	379.61
Temperature/K	100.00(13)
Crystal system	monoclinic
Space group	$P2_1$
a/Å	6.4481(4)
b/Å	16.6531(7)
c/Å	10.1178(6)
α/°	90
β/°	105.420(6)
γ/°	90
Volume/Å^3	1047.35(10)
Z	2
ρ_{calc}g/cm^3	1.204
μ/mm^{-1}	0.502
F(000)	420
Crystal size/mm^3	0.155 × 0.046 × 0.029
Radiation	Cu Kα (λ = 1.54184)
2Θ range for data collection/°	9.066 to 136.488
Index ranges	-6 ≤ h ≤ 7, -20 ≤ k ≤ 20, -12 ≤ l ≤ 12
Reflections collected	3721
Independent reflections	3721 [R_{int} = 0.095, R_{sigma} = 0.0122]
Data/restraints/parameters	3721/1/254
Goodness-of-fit on F^2	1.048
Final R indexes [I>=2σ (I)]	R_1 = 0.0878, wR_2 = 0.2420
Final R indexes [all data]	R_1 = 0.0928, wR_2 = 0.2480
Largest diff. peak/hole / e Å^{-3}	1.04/-0.24
Flack parameter	-1.4(10)

Table S-14 Fractional Atomic Coordinates (×10^{4}) and Equivalent Isotropic Displacement Parameters (Å^{2}×10^{3}) for amine **8g**. U_{eq} is defined as 1/3 of of the trace of the orthogonalised U_{IJ} tensor.

**Atom**	***x***	***y***	***z***	**U(eq)**
N1	2888(7)	4947(3)	4320(4)	27.8(10)
C1	3511(8)	4917(3)	5859(5)	25.9(11)
C2	4392(9)	5708(3)	6603(6)	31.2(12)
C3	1660(9)	4627(3)	6453(6)	34.0(13)
C4	5439(9)	4320(3)	6342(6)	32.6(12)
C5	5059(10)	5630(3)	8166(6)	35.7(13)
C6	3153(11)	5338(4)	8677(6)	38.7(14)
C7	2371(10)	4527(4)	8007(7)	35.5(14)
C8	4215(11)	3914(4)	8400(6)	38.2(14)
C9	6113(10)	4217(4)	7910(6)	36.9(14)
C10	6891(9)	5013(4)	8596(6)	34.4(13)
C11	1986(9)	5706(3)	3572(6)	26.3(11)
C12	62(9)	6069(3)	3972(6)	31.6(12)
C13	3795(9)	6344(3)	3705(6)	29.8(12)
C14	1252(10)	5541(3)	2008(6)	32.1(13)
C15	-1479(10)	6641(4)	1629(7)	39.6(14)
C16	414(10)	6300(3)	1161(6)	31.6(12)
C17	2216(10)	6926(3)	1383(6)	34.4(13)
C18	2951(9)	7109(3)	2897(6)	31.8(12)
C19	1085(10)	7454(4)	3395(6)	34.5(13)
C20	-729(10)	6844(4)	3154(6)	36.0(14)
C21	2143(9)	4150(3)	3724(6)	32.8(13)
C22	-278(12)	3902(4)	3145(9)	59(2)
C23	3172(15)	3764(4)	2654(7)	55(2)
C24	1037(13)	3640(4)	1351(7)	53.6(19)
C25	3431(17)	2871(5)	3063(10)	72(3)
C26	1256(15)	2553(5)	2880(8)	64(2)
C27	-284(15)	3253(5)	2155(10)	66(2)

Table S-15 Bond Lengths for compound **8g** (CCDC 2002570).

Atom	Atom	Length/Å	Atom	Atom	Length/Å
N1	C1	1.501(7)	C12	C20	1.544(8)
N1	C11	1.508(6)	C13	C18	1.534(7)
N1	C21	1.484(7)	C14	C16	1.541(7)
C1	C2	1.548(7)	C15	C16	1.531(9)
C1	C3	1.550(8)	C15	C20	1.527(9)
C1	C4	1.565(7)	C16	C17	1.532(8)
C2	C5	1.530(8)	C17	C18	1.509(8)
C3	C7	1.525(9)	C18	C19	1.534(9)
C4	C9	1.539(8)	C19	C20	1.519(9)
C5	C6	1.534(9)	C21	C22	1.570(9)
C5	C10	1.539(8)	C21	C23	1.551(9)
C6	C7	1.535(8)	C22	C27	1.473(12)
C7	C8	1.537(9)	C23	C24	1.647(12)
C8	C9	1.523(9)	C23	C25	1.542(11)
C9	C10	1.517(8)	C24	C27	1.473(12)
C11	C12	1.529(8)	C25	C26	1.464(14)
C11	C13	1.557(7)	C26	C27	1.581(12)
C11	C14	1.551(8)			

Table S-16 Bond Angles for compound **8g** (CCDC 2002570).

Atom	Atom	Atom	Angle/˚	Atom	Atom	Atom	Angle/˚
C1	N1	C11	120.6(4)	C14	C11	C13	103.1(4)
C21	N1	C1	111.2(4)	C11	C12	C20	111.3(5)
C21	N1	C11	120.3(4)	C18	C13	C11	111.7(4)
N1	C1	C2	116.0(4)	C16	C14	C11	112.9(4)
N1	C1	C3	113.0(4)	C20	C15	C16	109.3(5)
N1	C1	C4	108.5(4)	C15	C16	C14	108.8(5)
C2	C1	C3	107.3(4)	C15	C16	C17	109.8(5)
C2	C1	C4	103.3(4)	C17	C16	C14	109.6(4)
C3	C1	C4	108.1(5)	C18	C17	C16	107.9(5)
C5	C2	C1	113.4(5)	C13	C18	C19	109.5(5)
C7	C3	C1	112.5(5)	C17	C18	C13	110.1(5)
C9	C4	C1	112.1(5)	C17	C18	C19	110.3(4)
C2	C5	C6	110.2(5)	C20	C19	C18	109.6(5)
C2	C5	C10	109.4(5)	C15	C20	C12	109.1(5)
C6	C5	C10	108.5(5)	C19	C20	C12	110.0(5)
C5	C6	C7	109.2(5)	C19	C20	C15	109.5(5)
C3	C7	C6	109.5(5)	N1	C21	C22	124.7(5)
C3	C7	C8	109.8(5)	N1	C21	C23	120.1(5)
C6	C7	C8	109.2(5)	C23	C21	C22	101.4(6)
C9	C8	C7	109.2(5)	C27	C22	C21	105.6(7)
C8	C9	C4	110.0(5)	C21	C23	C24	100.9(6)
C10	C9	C4	110.0(5)	C25	C23	C21	104.4(6)
C10	C9	C8	109.7(5)	C25	C23	C24	95.8(6)
C9	C10	C5	108.6(4)	C27	C24	C23	95.5(6)
N1	C11	C12	115.5(4)	C26	C25	C23	106.5(7)
N1	C11	C13	110.4(4)	C25	C26	C27	104.7(6)
N1	C11	C14	109.7(4)	C22	C27	C24	99.0(6)
C12	C11	C13	110.4(4)	C22	C27	C26	109.8(7)
C12	C11	C14	106.9(4)	C24	C27	C26	101.2(7)

Details of the Single Crystal Structure Determination of Compound 8h (8-cyclohexyl-7,7,9,9-tetramethyl-1,4-dioxa-8-azaspiro[4.5]decane, CCDC 2002568)

Crystallization experiments at different temperatures and solvents were performed (methanol, ethanol, water/ethanol-mixture, isopropanol, formic acid ethyl ester, cooling of ethanolic solution to -10°C) and lead to large and seemingly high-quality crystals. However, all crystals were intrinsically disordered and all attempts to grow non-disordered crystals failed. Accordingly, the structure has been determined from a disordered crystal. A suitable crystal obtained from ethanol solution was measured on a Bruker SMART Platform diffractometer equipped with an Apex I-detector at 100 K. Using Olex2 [S-1], the structure was solved with the ShelXT [S-3] structure solution program using Intrinsic Phasing and refined with the ShelXL [S-5] refinement package using Least Squares minimisation. The molecules are distributed over four sites with equal occupancy. The refinement as a complete disordered molecule requires many parameters, leading to a poor data/parameter ratio. Therefore, several constraints (cyclohexyl-group described as a rigid goup) and restraints (chemically equivalent bond lengths were restrained to be equal with SADI and the displacement parameters were restrained with RIGU) were applied. The distance of the nitrogen atom N1 to plane defined by C11-C1A-C7 is 0.268(3) Å.

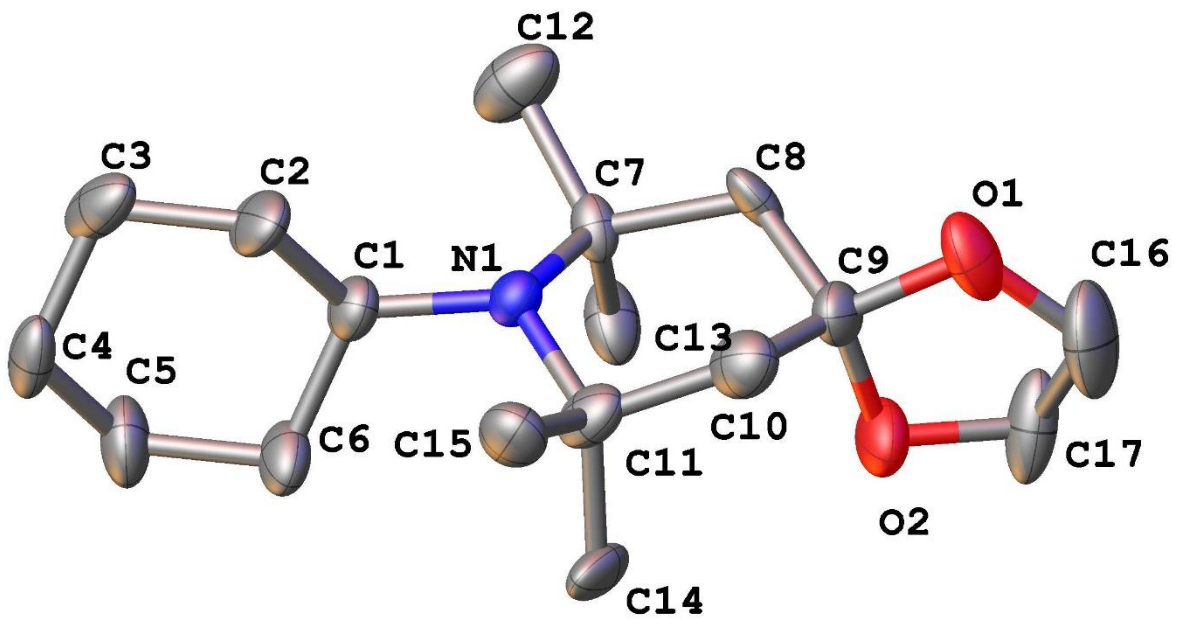

Figure S-9: View of one orientation of the *N*-cyclohexyl-tetramethylpiperidin molecule. The hydrogen atoms were omitted for clarity. Carbon atoms represented as grey oxygen atoms as red and nitrogen atoms as blue 50 % probability ellipsoids.

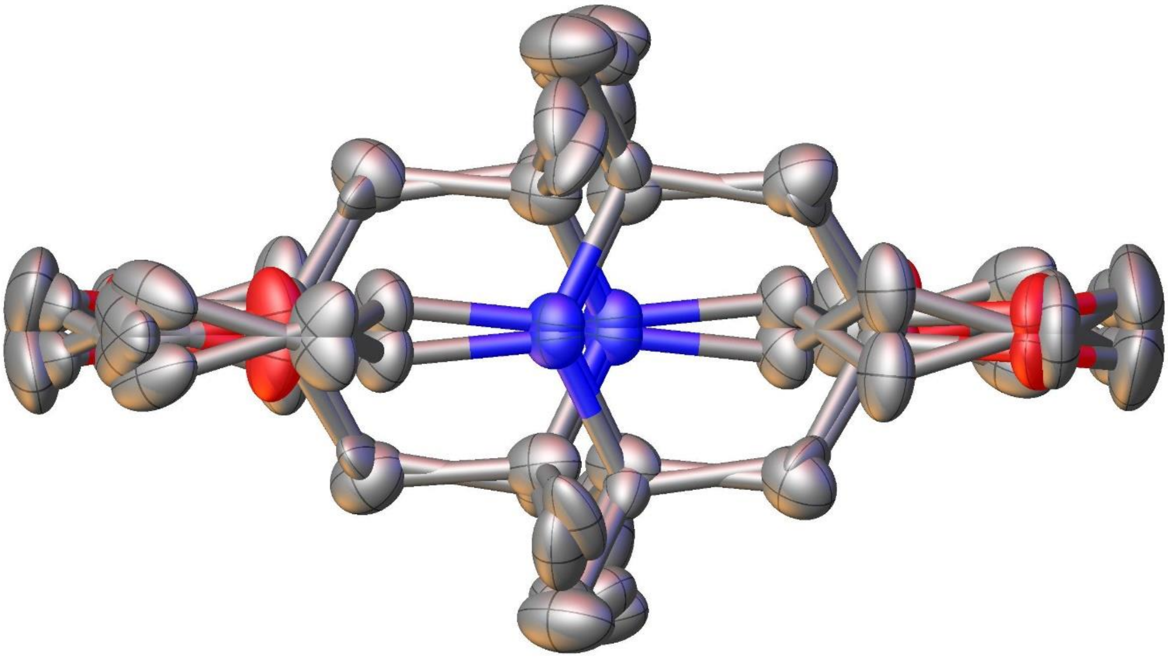

Figure S-10: Superposition of the four orientations of the 8-cyclohexyl-7,7,9,9-tetramethyl-1,4-dioxa-8-azaspiro[4.5]decane molecule. The hydrogen atoms were omitted for clarity. Carbon atoms represented as grey oxygen atoms as red and nitrogen atoms as blue 50 % probability ellipsoids.

Table S-17 Crystal data and structure refinement for **8h** (CCDC 2002568).

Identification code	all
Empirical formula	$C_{17}H_{31}NO_2$
Formula weight	281.43
Temperature/K	100
Crystal system	monoclinic
Space group	*C*2/*m*
a/Å	14.812(7)
b/Å	8.785(4)
c/Å	6.285(3)
α/°	90
β/°	104.877(6)
γ/°	90
Volume/Å^3	790.3(6)
Z	2
ρ_{calc}g/cm^3	1.183
μ/mm^{-1}	0.076
F(000)	312.0
Crystal size/mm^3	0.404 × 0.403 × 0.24
Radiation	MoKα (λ = 0.71073)
2Θ range for data collection/°	5.44 to 62.98
Index ranges	-21 ≤ h ≤ 20, -12 ≤ k ≤ 12, -9 ≤ l ≤ 8
Reflections collected	4638
Independent reflections	1297 [R_{int} = 0.0360, R_{sigma} = 0.0367]
Data/restraints/parameters	1297/182/171
Goodness-of-fit on F^2	1.060
Final R indexes [I>=2σ (I)]	R_1 = 0.0495, wR_2 = 0.1266
Final R indexes [all data]	R_1 = 0.0799, wR_2 = 0.1418
Largest diff. peak/hole / e Å^{-3}	0.10/-0.16

Table S-18 Fractional Atomic Coordinates (×10^4) and Equivalent Isotropic Displacement Parameters (Å^2×10^3) for amine **8h**. U_{eq} is defined as 1/3 of of the trace of the orthogonalised U_{IJ} tensor.

Atom	*x*	*y*	*z*	U(eq)
O1	7754(2)	5200(30)	6451(8)	49(3)
O2	6826(2)	5190(30)	8794(5)	49(3)
N1	4834.4(15)	4916(14)	4115(3)	27.7(8)
C5	2154.4(18)	4912(9)	3647(7)	53(5)
C1	3802.0(17)	4746(7)	3271(4)	34.9(14)
C2	3457(3)	5111(10)	828(4)	47.1(15)
C3	2449(3)	4596(11)	-94(5)	49.5(19)
C4	1808.8(18)	5277(11)	1203(7)	41(3)
C6	3162(2)	5423(5)	4571(4)	43.7(15)
C7	5269(6)	3445(13)	4992(16)	28.4(17)
C8	6343(5)	3698(13)	5420(20)	31.4(19)
C9	6800(2)	5129(15)	6494(6)	36.4(14)
C10	6308(7)	6494(16)	5290(20)	44(2)
C11	5245(7)	6358(14)	5167(18)	39(2)
C12	4922(12)	2169(18)	3300(30)	55(3)
C13	5110(11)	2910(20)	7199(19)	44(4)
C14	5020(13)	6800(20)	7350(20)	44(3)
C15	4913(11)	7697(17)	3580(30)	43(2)
C16	8320(5)	5330(30)	8557(12)	75(5)
C17	7717(4)	4870(20)	10022(11)	59(3)

Table S-19 Bond Lengths for **8h** (CCDC 2002568).

Atom	Atom	Length/Å	Atom	Atom	Length/Å
O1	C9	1.421(5)	C2	C3	1.5249
O1	C16	1.378(5)	C3	C4	1.5226
O2	C9	1.437(5)	C7	C8	1.560(4)
O2	C17	1.378(5)	C7	C12	1.539(3)
N1	C1	1.492(4)	C7	C13	1.539(3)
N1	C7	1.486(3)	C8	C9	1.503(3)
N1	C11	1.485(3)	C9	C10	1.503(3)
C5	C4	1.5228	C10	C11	1.560(4)
C5	C6	1.5235	C11	C14	1.539(3)
C1	C2	1.5229	C11	C15	1.539(3)
C1	C6	1.5220	C16	C17	1.492(11)

Table S-20 Bond Angles for **8h** (CCDC 2002568).

Atom	Atom	Atom	Angle/˚	Atom	Atom	Atom	Angle/˚
C16	O1	C9	110.4(4)	C13	C7	C12	107.3(11)
C17	O2	C9	109.2(5)	C9	C8	C7	121.2(7)
C7	N1	C1	110.6(7)	O1	C9	O2	104.3(3)
C7	N1	C11	119.2(2)	O1	C9	C8	111.4(11)
C11	N1	C1	120.6(7)	O1	C9	C10	108.1(11)
C4	C5	C6	111.4	O2	C9	C8	112.2(12)
N1	C1	C2	112.7(3)	O2	C9	C10	110.9(12)
N1	C1	C6	119.5(3)	C8	C9	C10	109.7(3)
C6	C1	C2	111.4	C9	C10	C11	108.5(8)
C1	C2	C3	111.3	N1	C11	C10	112.4(8)
C4	C3	C2	111.3	N1	C11	C14	117.0(10)
C3	C4	C5	111.3	N1	C11	C15	109.6(11)
C1	C6	C5	111.4	C14	C11	C10	112.4(12)
N1	C7	C8	105.4(6)	C14	C11	C15	106.1(11)
N1	C7	C12	109.9(10)	C15	C11	C10	97.2(10)
N1	C7	C13	116.7(9)	O1	C16	C17	104.9(7)
C12	C7	C8	111.6(10)	O2	C17	C16	103.8(7)
C13	C7	C8	105.9(10)				

Details of the Single Crystal Structure Determination of Compound 8i (8-neopentyl-7,7,9,9-tetramethyl-1,4-dioxa-8-azaspiro[4.5]decane, CCDC 2004218)

The compound **8i** is a liquid at room temperature. A small drop of the compound was sealed in a 0.5 mm Mark-tube, mounted on the diffractometer and slowly cooled to 100 K. The compound forms a supercooled liquid and the diffraction pattern shows only very broad powder rings at low angles, typical for amorphous compounds. After tempering for one night at 218 K, the reflections in diffraction pattern became more pronounced with smaller line width, still diffracting to a maximum of about 8° in 2Theta. Slow warming to 298 K and subsequent oszillation of the temperature lead to crystallization of sufficiently large crystals in some areas in the Mark tube. A well diffracting area was chosen and a data set of a full sphere up to 0.78 Å resolution was collected at 100 K. The diffraction pattern showed strong contributions of at least 4 individuals. Nevertheless, it was possible to index about 30% of the reflections belonging to the largest individual with a monoclinic unit cell and the data reduction was performed. The resulting figures of merit are surprisingly good, meaning that there are only few overlapping reflections with the other individuals. The unit cell contains two crystallographically independent molecules. The distance of the atom N1 from the plane defined by the neighboring carbon atoms C6-C12-C1 is 0.3414(16) Å. The corresponding distance in the second molecule of the atom N2 from the plane defined by C28-C17-C22 is 0.3450(17) Å.

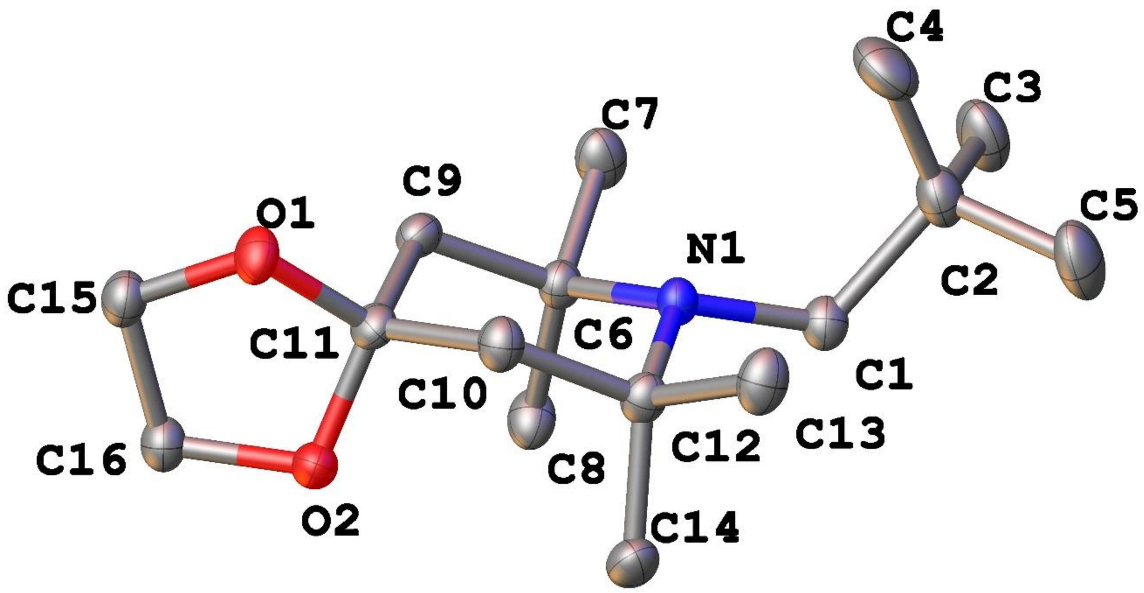

Figure S-11: View of 8-neopentyl-7,7,9,9-tetramethyl-1,4-dioxa-8-azaspiro[4.5]decane compound 8i. Carbon atoms represented as grey, oxygen atoms as red and nitrogen atoms as blue 50 % probability ellipsoids.

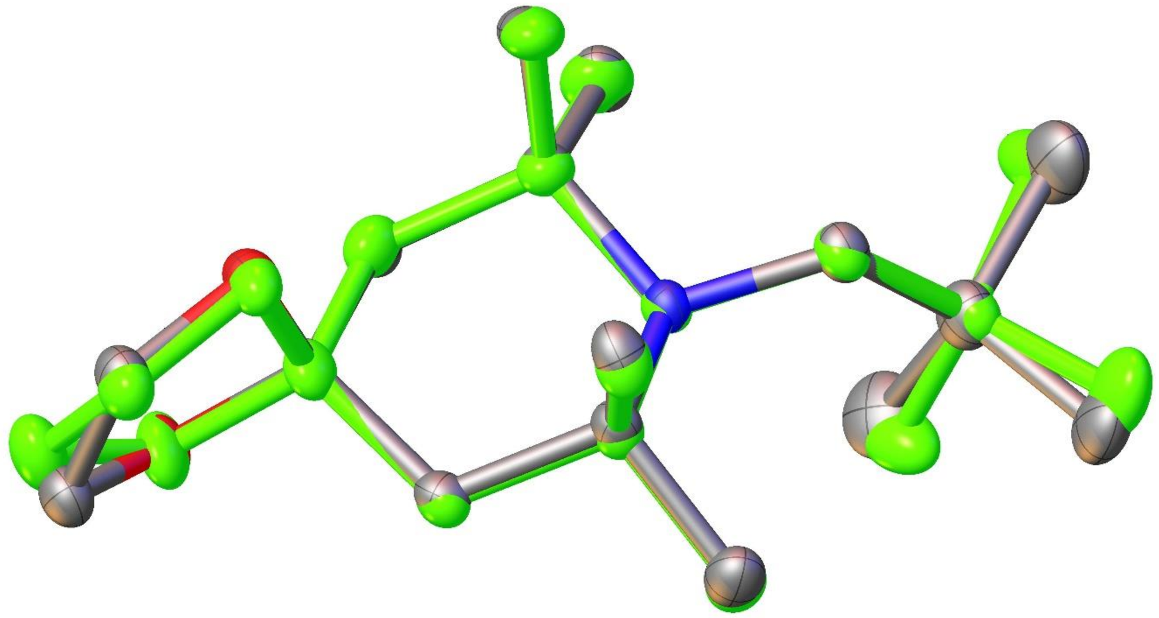

Figure S-12: An overlay of the two crystallographically independent molecules in the crystal structure of 8i. Carbon atoms represented as grey, oxygen atoms as red and nitrogen atoms as blue 50 % probability ellipsoids. The second crystallographically independent molecule is shown in green.

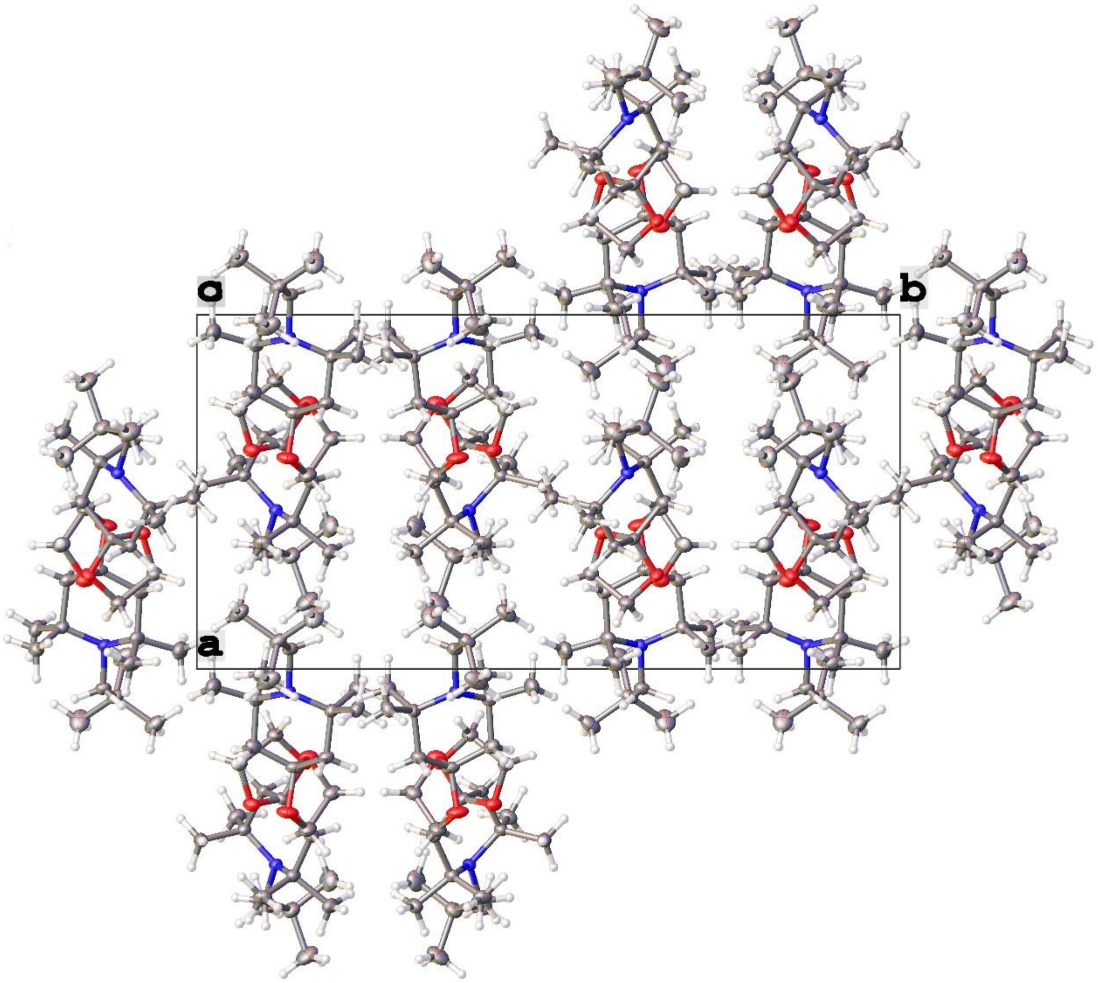

Figure S-13: View along the crystallographic c direction of the packing of 8i. Carbon atoms represented as grey, oxygen atoms as red and nitrogen atoms as blue 50 % probability ellipsoids.

Table S-21 Crystal data and structure refinement for compound **8i** (CCDC 2004218)

Identification code	amin15r05d
Empirical formula	$C_{16}H_{31}NO_2$
Formula weight	269.42
Temperature/K	99.97(13)
Crystal system	monoclinic
Space group	$P2_1/c$
a/Å	12.6810(15)
b/Å	22.441(2)
c/Å	12.6112(15)
α/°	90
β/°	116.987(15)
γ/°	90
Volume/Å^3	3198.0(7)
Z	8
ρ_{calc}g/cm^3	1.119
μ/mm^{-1}	0.072
F(000)	1200.0
Crystal size/mm^3	0.5 × 0.5 × 0.5
Radiation	Mo Kα (λ = 0.71073)
2Θ range for data collection/°	3.604 to 54.202
Index ranges	-16 ≤ h ≤ 16, -28 ≤ k ≤ 28, -16 ≤ l ≤ 16
Reflections collected	54266
Independent reflections	7051 [R_{int} = 0.0686, R_{sigma} = 0.0478]
Data/restraints/parameters	7051/0/357
Goodness-of-fit on F^2	1.026
Final R indexes [I>=2σ (I)]	R_1 = 0.0548, wR_2 = 0.1147
Final R indexes [all data]	R_1 = 0.0830, wR_2 = 0.1284
Largest diff. peak/hole / e Å^{-3}	0.23/-0.22

Table S-22 Fractional Atomic Coordinates (×10^4) and Equivalent Isotropic Displacement Parameters (Å^2×10^3) for amine **8i**. U_{eq} is defined as 1/3 of of the trace of the orthogonalised U_{IJ} tensor.

Atom	*x*	*y*	*z*	U(eq)
O1	7352.9(10)	6539.1(5)	6622.0(11)	25.2(3)
N1	4473.9(11)	6124.2(6)	7257.6(12)	18.8(3)
C1	3443.6(14)	5963.3(7)	7446.3(15)	22.9(4)
O2	6165.2(10)	5778.5(5)	5611.8(10)	24.5(3)
C2	3192.2(16)	6346.7(8)	8355.7(17)	30.4(4)
C3	3314.4(18)	5951.1(10)	9399.9(18)	39.5(5)
C4	4019(2)	6880.5(9)	8860.6(19)	42.1(5)
C5	1905.2(18)	6567.7(10)	7731(2)	43.2(5)
C6	5454.2(13)	5674.8(7)	7731.6(14)	20.0(3)
C7	5823.7(15)	5569.4(9)	9056.6(16)	29.0(4)
C8	5161.7(15)	5057.3(7)	7130.8(17)	26.6(4)
C9	6544.4(13)	5947.7(7)	7680.6(14)	20.4(3)
C10	6331.9(14)	6212.8(7)	6497.5(15)	20.8(4)
C11	5289.1(14)	6633.2(8)	6060.3(16)	23.5(4)
C12	4160.2(14)	6373.5(7)	6053.4(15)	21.5(4)
C13	3311.5(15)	6900.7(8)	5805.0(17)	29.8(4)
C14	3558.0(15)	5935.5(9)	5010.4(16)	29.4(4)
C15	8132.5(15)	6112.6(8)	6498.4(16)	26.0(4)
C16	7313.3(14)	5605.7(8)	5779.7(16)	25.3(4)
N2	9338.1(11)	6310.9(6)	2198.1(12)	20.0(3)
O3	5948.6(10)	6291.5(6)	1995.2(11)	35.3(3)
O4	7498.3(10)	6587.7(6)	3722.0(10)	28.4(3)
C17	10556.6(14)	6331.0(8)	2337.4(15)	23.4(4)
C18	10790.7(15)	6157.8(8)	1248.1(16)	26.4(4)
C19	11572.4(17)	5599.4(9)	1551.0(17)	36.1(5)
C20	11467(2)	6664.2(10)	1026(2)	46.0(6)
C21	9667.0(18)	6028.0(10)	101.0(17)	40.8(5)
C22	9142.9(14)	5847.3(8)	2936.2(15)	24.3(4)
C23	9485.2(17)	5236.2(8)	2639.4(18)	35.2(5)
C24	9853.3(16)	5931.6(9)	4300.9(16)	32.0(4)
C25	7811.2(15)	5809.2(8)	2576.8(16)	28.0(4)
C26	7207.3(14)	6389.5(8)	2545.1(15)	26.2(4)
C27	7497.5(14)	6841.7(8)	1827.9(15)	23.9(4)
C28	8821.2(14)	6914.5(7)	2174.0(15)	21.6(4)
C29	8867.5(16)	7282.6(8)	1172.3(16)	28.7(4)
C30	9444.7(15)	7281.9(8)	3329.7(16)	27.5(4)

Table S-22 Fractional Atomic Coordinates (×10^4) and Equivalent Isotropic Displacement Parameters (Å^2×10^3) for amine **8i**. U_{eq} is defined as 1/3 of of the trace of the orthogonalised U_{IJ} tensor.

**Atom**	***x***	***y***	***z***	**U(eq)**
C31	5473.1(16)	6576.5(9)	2691.8(19)	36.0(5)
C32	6498.1(15)	6923.9(9)	3622.3(16)	29.8(4)

Table S-23 Bond Lengths for compound **8i** (CCDC 2004218)

**Atom**	**Atom**	**Length/Å**	**Atom**	**Atom**	**Length/Å**
O1	C10	1.4328(18)	N2	C17	1.475(2)
O1	C15	1.434(2)	N2	C22	1.490(2)
N1	C1	1.4751(19)	N2	C28	1.499(2)
N1	C6	1.498(2)	O3	C26	1.440(2)
N1	C12	1.493(2)	O3	C31	1.423(2)
C1	C2	1.578(2)	O4	C26	1.428(2)
O2	C10	1.4242(19)	O4	C32	1.431(2)
O2	C16	1.4265(19)	C17	C18	1.580(2)
C2	C3	1.537(3)	C18	C19	1.535(2)
C2	C4	1.528(3)	C18	C20	1.525(3)
C2	C5	1.537(3)	C18	C21	1.529(3)
C6	C7	1.535(2)	C22	C23	1.535(3)
C6	C8	1.542(2)	C22	C24	1.550(2)
C6	C9	1.540(2)	C22	C25	1.539(2)
C9	C10	1.513(2)	C25	C26	1.502(2)
C10	C11	1.510(2)	C26	C27	1.512(2)
C11	C12	1.542(2)	C27	C28	1.540(2)
C12	C13	1.533(2)	C28	C29	1.533(2)
C12	C14	1.539(2)	C28	C30	1.544(2)
C15	C16	1.530(2)	C31	C32	1.513(3)

Table S-24 Bond Angles for compound **8i** (CCDC 2004218)

Atom	Atom	Atom	Angle/˚	Atom	Atom	Atom	Angle/˚
C10	O1	C15	106.27(12)	C17	N2	C22	114.16(12)
C1	N1	C6	113.96(12)	C17	N2	C28	113.56(13)
C1	N1	C12	114.15(12)	C22	N2	C28	116.65(12)
C12	N1	C6	116.60(12)	C31	O3	C26	108.77(14)
N1	C1	C2	118.02(14)	C26	O4	C32	105.79(13)
C10	O2	C16	106.94(12)	N2	C17	C18	119.28(14)
C3	C2	C1	109.45(15)	C19	C18	C17	109.84(14)
C3	C2	C5	107.22(15)	C20	C18	C17	108.65(15)
C4	C2	C1	113.82(15)	C20	C18	C19	107.49(15)
C4	C2	C3	108.22(17)	C20	C18	C21	109.19(17)
C4	C2	C5	109.19(17)	C21	C18	C17	114.04(14)
C5	C2	C1	108.75(15)	C21	C18	C19	107.43(16)
N1	C6	C7	109.03(13)	N2	C22	C23	109.02(14)
N1	C6	C8	115.96(13)	N2	C22	C24	115.54(14)
N1	C6	C9	108.28(13)	N2	C22	C25	109.03(13)
C7	C6	C8	106.44(14)	C23	C22	C24	107.07(15)
C7	C6	C9	105.69(13)	C23	C22	C25	105.80(15)
C9	C6	C8	110.94(13)	C25	C22	C24	109.93(14)
C10	C9	C6	115.86(13)	C26	C25	C22	115.95(14)
O1	C10	C9	110.20(13)	O3	C26	C25	108.71(14)
O1	C10	C11	108.59(13)	O3	C26	C27	109.39(14)
O2	C10	O1	103.94(12)	O4	C26	O3	104.79(13)
O2	C10	C9	113.64(13)	O4	C26	C25	110.75(14)
O2	C10	C11	110.33(13)	O4	C26	C27	113.23(14)
C11	C10	C9	109.90(13)	C25	C26	C27	109.77(14)
C10	C11	C12	115.02(14)	C26	C27	C28	115.58(14)
N1	C12	C11	108.53(13)	N2	C28	C27	108.69(13)
N1	C12	C13	109.59(13)	N2	C28	C29	108.90(13)
N1	C12	C14	115.46(14)	N2	C28	C30	115.56(14)
C13	C12	C11	105.87(13)	C27	C28	C30	110.47(13)
C13	C12	C14	106.75(14)	C29	C28	C27	105.56(13)
C14	C12	C11	110.22(14)	C29	C28	C30	107.19(14)
O1	C15	C16	104.15(13)	O3	C31	C32	104.76(14)
O2	C16	C15	104.99(13)	O4	C32	C31	102.22(14)

Compounds 8b, 8c, 8d and 8j, for which no crystals with sufficient long range order were obtained:

8b: The compound solidifies below about 30 °C and forms a plastic phase of probably hexagonal symmetry. Due to the disorder, no diffraction intensities above about 4° in 2θ, which corresponds to a resolution of 10 Å, can be observed. Below about 270 K a solid-solid phase transition takes place and the sample becomes polycrystalline. Tempering of the sample slightly below the phase transition temperature for several hours did not lead to the formation of single crystals. Attempts to grow single crystals from a diethyl ether solution by slow evaporation of the solvent (2 weeks) at room temperature and at -20 °C was not successful.

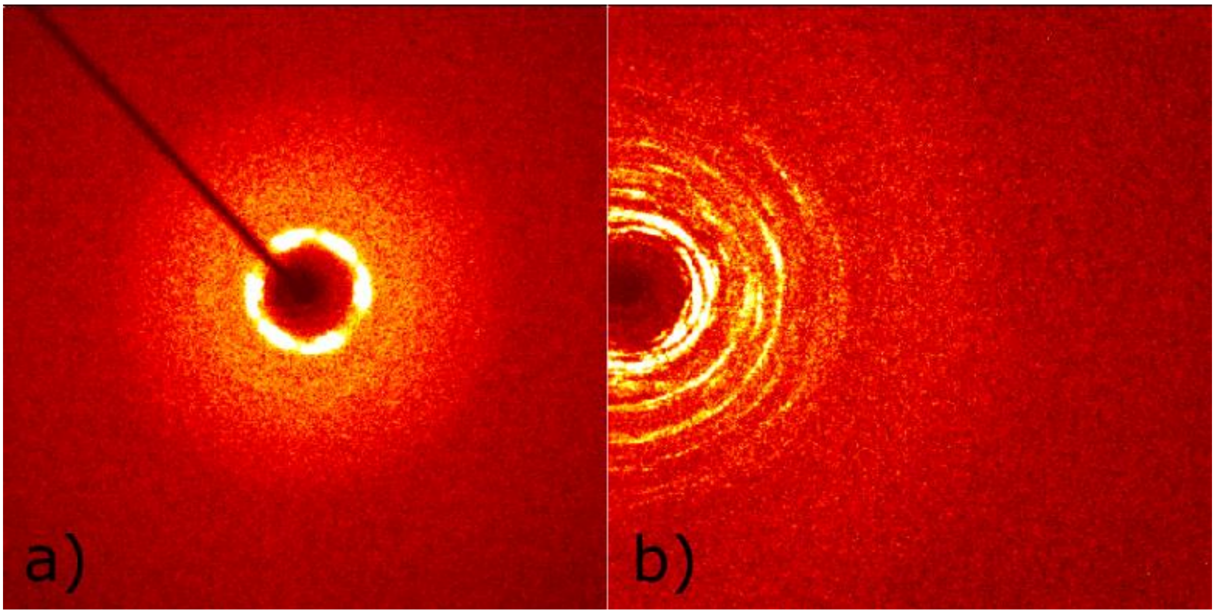

Figure S-14: **(a) Diffraction pattern of the plastic phase of compound 8b at 275 K, probably with hexagonal symmetry. No high angle reflections are visible. (b) Diffraction pattern of compound 8b, recorded at 100 K of the polycrystalline phase, which forms below about 270 K**

8c: melts at about 60 °C and forms a plastic phase when cooled. Attempts to grow ordered crystals at low temperature from a hexane solution at low temperature or by evaporating of the solvent were not successful.

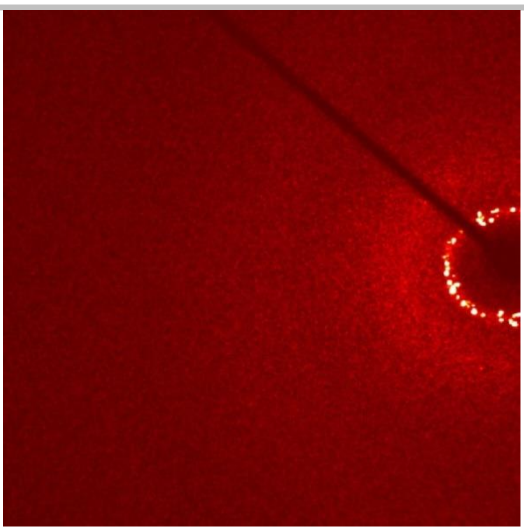

Figure S-15: **Diffraction pattern of the plastic phase at 120 K of compound 8c.**

8d: The compound crystallizes as very weakly diffracting thin platelets. Reflections were observed up to a maximum diffraction angle of about 6.8° in 2θ, corresponding to a resolution of only about 6 Å, and no further measurements were performed.

Tri-*tert*-butylmethanol: The compound crystallizes in a hexagonal unit cell with a = 14.40527(9) Å and c = 25.9741(1) Å (V = 4667.82(5) Å^3) in the space group $P6_3$. There are 5 crystallographically independent molecules in the unit cell, all of them showing strong positional disorder, and one water molecule. Because of the strong disorder (and/or twinning) and the rather unsatisfactory R-values, accurate bond distances and precise geometric details cannot be obtained from this model.

Table S-25: CCCN Angles of the Crystal and Gas Phase of compounds **8a** and **8e**–**i** using different functionals and post-Hartree-Fock methods compared to experiment. The extrapolated MP2 and SCS-MP2 [S-15] aug-cc-pV(D,T)Z results were abbreviated with aDTZ.

CCCN Angle	cut-off/	**8a**		**8e**		**8f**		**8g**		**8h**		**8i**	
	basis set	Crystal	Gas Phase	Crystal	Gas Phase	Crystal	Gas Phase	Crystal	Gas Phase	Crystal	Gas Phase	Crystal	Gas Phase
Experiment		17.9		17.5		16.2		16.7		18.5		25	
		20.7		17.7		18.6							
				19.7		18.7							
				19.9		19.6							
BLYP+D3	900	17.9	16.9	17.5	18.4	15.4	16.6	15.8	15.9	19.6	18.6	23.2	23.8
		20		17.6		17.5		18.5				24	
				19.7								24.3	
				20.8								25.2	
BLYP+D3	1200	17.3	16.4	17.3	18.4	17.3	19	15.7	15.8			23.2	23.7
		19.8		17.5		17.3		18				24	
				19.4		18						24.1	
				19.6		21.8						25	
optB88-vdW	1200	17.9	16.6	17.3	18.6	15.6	20	16.1	16	17.8	17.2	23.4	25
		20.4		18.6		17.9		18.6				24.2	
				19.4		17.9						24.3	
				19.9		18.8						25.4	
PBED3	900	17.7	17.1	16.5	18.4	15.5	20.1	18.5	15.8	19.6	18.8	23.6	24.9
		20.2		16.6		15.5						24.2	
				18.8		17.8						24.4	
				18.8		17.8							
PBETS	900	16.2	15.4	16.5	17.6	13.6	19	17.3	15.2	19.7	18.4	22.8	24.5
		16.5		16.6		13.6		17.7				23.5	
		18.6		18.8		15.8						24	
				18.8		15.8						25.2	
RPBED3	900	17.9	18.2	18.1	19.5	16.7	21.3	19.7	16.8	20.3	19.5	24.6	25
		20.7		18.3		19.2						25.1	
				20.3		19.2						24.4	
				20.5		20.2						26.3	
vdW-DF2	1200	17.6	17.1	17.3	18.7	15.2	20	18.2	15.4	16.4	17	23.4	24.8
		20.1		17.5		15.2						24.1	
				19.7		17.6						24.6	
				20.3		17.6						25.4	
B3LYPD3	TZVPPD		16.3		18.8		19.2		17.1		15.9		24
MP2	aDTZ		19.4		22.3		23		15.6				26.4
SCS-MP2	aDTZ		18.8		20.5		22.4		19				25.2

Table S-26: Pyramidal Distance in Ångstrom of the Crystal and Gas Phase of compounds **8a** and **8e**–**i** using different functionals and post-Hartree-Fock methods compared to experiment. The extrapolated MP2 and SCS-MP2 aug-cc-pV(D,T)Z results were abbreviated with aDTZ.

Pyramidal	cut-off/	8a		8e		8f		8g		8h		8i	
Distance	basis set	Crystal	Gas Phase	Crystal	Gas Phase	Crystal	Gas Phase	Crystal	Gas Phase	Crystal	Gas Phase	Crystal	Gas Phase
Experiment		0.254		0.259		0.241		0.243		0.268		0.341	
												0.345	
BLYP+D3	900	0.245	0.23	0.258	0.241	0.223	0.248	0.232	0.237	0.254	0.239	0.316	0.325
				0.26								0.331	
BLYP+D3	1200	0.241	0.229	0.255	0.241	0.223	0.247	0.231	0.236			0.317	0.325
				0.257								0.329	
optB88-vdW	1200	0.248	0.23	0.253	0.242	0.23	0.247	0.235	0.237	0.25	0.242	0.317	0.33
				0.259								0.334	
PBED3	900	0.245	0.232	0.25	0.24	0.228	0.246	0.232	0.234	0.251	0.24	0.319	0.328
				0.256								0.331	
PBETS	900	0.223	0.207	0.242	0.228	0.2	0.231	0.22	0.226	0.251	0.239	0.307	0.323
				0.244								0.328	
RPBED3	900	0.25	0.245	0.264	0.255	0.247	0.26	0.249	0.249	0.26	0.25	0.334	0.34
				0.268								0.345	
vdW-DF2	1200	0.248	0.233	0.257	0.248	0.229	0.25	0.236	0.235	0.232	0.243	0.317	0.332
				0.262								0.334	
B3LYPD3	TZVPPD		0.219		0.231		0.25		0.208		0.224		0.317
MP2	aDTZ		0.264		0.275		0.281		0.231				0.346
SCS-MP2	aDTZ		0.255		0.269		0.275		0.232				0.344
DFT average		0.24317	0.2295	0.254	0.24233333	0.22617	0.247	0.234	0.23633333	0.24967	0.24216667	0.31833333	0.32966667

Table S-27: CCCN Angles of the Crystal and Gas Phase of trimethylamine, triethylamine, tripropylamine and triisopropylamine using different functionals and post-Hartree-Fock methods compared to experiment.

CCCN Angle	Trimethylamine				Triethylamine	Tripropylamine	Triisopropylamine	
	Crystal 1	Crystal 2	Crystal 3	Gas Phase	Gas Phase	Gas Phase	Crystal	Gas Phase
Experiment	33.4	33	34.1				22	
		34.8	34.5					
		35.3						
BLYP+D3/TZVPPD				31.9	25.7			
PBED3/TZVPPD				32.3	29.5			
PBED3/900 eV	32.8	32.6	32.7				2	
		32.8	33					
		32.9						
B3LYPD3/TZVPPD				31.8	25.8	30.9		15.8
MP2/aDTZ				34.6	30.1			20.6
SCS-MP2/ADTZ				34.3	29.7			19.9

For triisopropylamine, all CCCN angles in the solid are below 3 degrees for the following functionals: PBE+MBD [S-16], PBE0 [S-17]+MBD, PBE+TS, B3LYP+D3, BLYP+D3, as well as using an expanded cell volume for PBE+D3.

Table S-28: Pyramidal Distance in Ångstrom of the Crystal and Gas Phase of trimethylamine, triethylamine, tripropylamine and triisopropylamine using different functionals and post-Hartree-Fock methods compared to experiment.

Pyramidal	Trimethylamine				Triethylamine	Tripropylamine	Triisopropylamine	
Distance	Crystal 1	Crystal 2	Crystal 3	Gas Phase	Gas Phase	Gas Phase	Crystal	Gas Phase
Experiment	0.454	0.474	0.469				0.291	
BLYP+D3/TZVPPD				0.436	0.346			
PBED3/TZVPPD				0.438	0.391			
PBED3/900 eV	0.447	0.446	0.449				0.03	
B3LYPD3/TZVPPD				0.43	0.424	0.42		0.204
MP2/aDTZ				0.472	0.405			0.269
SCS-MP2/ADTZ				0.469	0.4			0.26

For triisopropylamine, all pyramidal distances in the solid are below 0.04 Ångstrom for the following functionals: PBE+MBD [S-16], PBE0 [S-17]+MBD, PBE+TS, B3LYP+D3, BLYP+D3, as well as using an expanded cell volume for PBE+D3.

Table S-29: Raw Data of the different gas phase conformers of Compounds **8a–8d**.

Configurations		**8a**	**8b**	**8c**	**8d**
Raw Data	0	-449.5401813	-488.8247225	-528.1262762	-567.4259047
Energy in Hartree	0.planar	-449.5378466	-488.8196531	-528.1222958	-567.4224157
B3LYP+D3/TZVPPD	1	-449.5401749	-488.8246989	-528.1261394	-567.4258854
	2	-449.5386511	-488.8202213	-528.1261355	-567.4263645
	3	-449.5386518	-488.8201908	-528.1242715	-567.4263583
	4	-449.5301682	-488.8162751	-528.1242774	-567.4248826
	5	-449.5301678	-488.8162584	-528.1235746	-567.4248906
	6			-528.1235778	-567.4245727
	7			-528.123173	-567.424578
	8			-528.1216164	-567.4232096
	9				
	crystal	-449.5401738			
Raw Data	0	-449.5019335	-488.7804589	-528.0765899	-567.3715808
B3LYP/TZVPPD	0.planar	-449.5002442	-488.7761607	-528.0728126	-567.3683702

Table S-30: Raw Data of the different gas phase conformers of Compounds **8e–8j**.

Configurations		**8e**	**8f**	**8g**	**8h**	**8i**	**8j**
Raw Data	0	-721.0334849	-1069.946787	-1108.01877	-871.4809359	-833.3826007	-605.5472708
Energy in Hartree	0.planar	-721.0295862	-1069.942206	-1108.015467	-871.4758202	-833.373516	-605.5380359
B3LYP+D3/TZVPPD	1	-721.0334848	-1069.946768		-871.4809375		-605.5470881
	2	-721.0318025	-1069.943083		-871.4809549		-605.5455983
	3	-721.0318153	-1069.937414		-871.4809526		-605.54244
	4	-721.028538			-871.4782978		-605.5424364
	5	-721.0285277			-871.478278		-605.5411674
	6	-721.0287322			-871.4770469		-605.5411715
	7	-721.0287488			-871.4770203		-605.5403965
	8				-871.4721265		-605.5403882
	9				-871.472054		-605.5390128
	crystal	-721.0334963	-1069.946742	-1108.018344			
Raw Data	0	-720.9681341	-1069.848823	-1107.918198	-871.4131882	-833.3159237	-605.4912091
B3LYP/TZVPPD	0.planar	-720.964941	-1069.845191	-1107.915771	-871.4088323	-833.3057914	-605.4809533

Table S-31: Relative energy differences and pyramidal distances in Ångstrom of the different gas phase conformers of Compounds **8a**–**8d**.

Configurations		8a		8b		8c		8d	
		Energy	N CCC Plane	Energy	N CCC Plane	Energy	N CCC Plane	Energy	N CCC Plane
B3LYP+D3/TZVPPD	0	0.0	0.221	0.0	0.229	0.0	0.226	1.2	0.214
Energy in kJ/mol	0.planar	6.1	0	13.3	0	10.5	0	10.4	0
Distance in A	1	0.0	0.221	0.1	0.229	0.4	0.226	1.3	0.215
	2	4.0	0.202	11.8	0.072	0.4	0.23	0.0	0.256
	3	4.0	0.203	11.9	0.069	5.3	0.214	0.0	0.257
	4	26.3	0.15	22.2	0.212	5.2	0.214	3.9	0.229
	5	26.3	0.148	22.2	0.207	7.1	0.225	3.9	0.23
	6					7.1	0.224	4.7	0.214
	7					8.1	0.217	4.7	0.213
	8					12.2	0.228	8.3	0.204
	9								
	crystal	0.0	0.219						
B3LYP/TZVPPD	0	0.0		0.0		0.0		0.0	
Energy in kJ/mol	0.planar	4.4		11.3		9.9		8.4	

Table S-32: Relative energy differences and pyramidal distances in Ångstrom of the different gas phase conformers of Compounds **8e**–**8j**.

Configurations		8e		8f		8g		8h		8i		8j	
		Energy	N CCC Plane	Energy	N CCC Plane	Energy	N CCC Plane	Energy	N CCC Plane	Energy	N CCC Plane	Energy	N CCC Plane
B3LYP+D3/TZVPPD	0	0.0	0.232	0.0	0.233	0.0	0.208	0.0	0.223	0.0	0.317	0.0	0.309
Energy in kJ/mol	0.planar	10.2		12.0		8.7		13.4		23.9		24.2	0
Distance in A	1	0.0	0.229	0.0	0.233			0.0				0.5	0.316
	2	4.4	0.202	9.7	0.092			0.0				4.4	0.353
	3	4.4	0.238	24.6	0.216			0.0				12.7	0.207
	4	13.0	0.1					6.9	0.086			12.7	0.208
	5	13.0	0.101					7.0				16.0	0.292
	6	12.5	0.075					10.2	0.212			16.0	0.292
	7	12.4						10.3				18.0	0.007
	8							23.1	0.104			18.1	0.005
	9							23.3				21.7	0.338
	crystal	0.0	0.231	0.1	0.25	1.1				0			
B3LYP/TZVPPD	0	0.0		0.0		0.0		0.0		0.0		0.0	
Energy in kJ/mol	0.planar	8.4		9.5		6.4		11.4		26.6		26.9	

Table S-33: Raw Data of the different gas phase conformers of trimethylamine, triethylamine, tripropylamine and triisopropylamine.

B3LYP+D3/TZVPPD		triisopropylamine	trimethylamine	triethylamine	tripropylamine
Raw Data	0	-410.2470215	-174.43104	-292.339867	-410.2471219
Energy in Hartree	0.planar	-410.2461162	-174.4191399	-292.3341537	-410.2422868
	1	-410.241195		-292.3398681	-410.2471195
	2	-410.2411935		-292.3390535	-410.2476063
	3	-410.2410536		-292.3390901	-410.2476199
	4	-410.2410598		-292.3386479	-410.2475554
	5	-410.2371708		-292.3370047	-410.2483282
	6	-410.237166		-292.337002	-410.2465875
	7				-410.2465929
	8				-410.2466624
	9				-410.246558

Table S-34: Relative energy differences and pyramidal distances in Ångstrom of the different gas phase conformers of trimethylamine, triethylamine, tripropylamine and triisopropylamine.

Configurations		triisopropylamine		trimethylamine		triethylamine		tripropylamine	
		Energy	N CCC Plane	Energy	N CCC Plane	Energy	N CCC Plane	Energy	N CCC Plane
B3LYP+D3/TZVPPD	0	0.0	0.2	0.0	0.43	0.0	0.424	3.2	0.42
Energy in kJ/mol	0.planar	2.4	0	31.2		15.0	0	15.9	0
Distance in A	1	15.3	0.33			0.0	0.423	3.2	0.419
	2	15.3	0.33			2.1	0.384	1.9	0.415
	3	15.7	0.212			2.0	0.388	1.9	0.412
	4	15.7	0.211			3.2	0.388	2.0	0.414
	5	25.9	0.287			7.5	0.301	0.0	0.411
	6	25.9	0.286			7.5	0.301	4.6	0.37
	7							4.6	0.374
	8							4.4	0.373
	9							4.6	0.37

Table S-35: Additional calculations on planar and non-planar structures of trimethylamine, triethylamine, tripropylamine and triisopropylamine.

		triisopropylamine	trimethylamine	triethylamine	tripropylamine
B3LYP/TZVPPD	0	0	0	0	0
Energy in kJ/mol	0.planar	1.0	29.1	17.9	15.2
Raw Data	0	-410.2149435	-174.4244438	-292.3240477	-410.2224014
Energy in Hartree	0.planar	-410.2145521	-174.4133433	-292.3172372	-410.216602
SCS-MP2/DTZ	0	0			
Energy in kJ/mol	0.planar	5.6			
Raw Data	0	-409.6110032			
Energy in Hartree	0.planar	-409.6088618			
MP2/DTZ	0	0			
Energy in kJ/mol	0.planar	6.5			
Raw Data	0	-409.5971916			
Energy in Hartree	0.planar	-409.5947022			
B3LYP+D3/TZVPPD	0	0			
+ZPE in kJ/mol	0.planar	-0.6			
Raw Data	0	-409.9577209			
Energy in Hartree	0.planar	-409.9579553			
B3LYP+D3/TZVPPD	0	0			
ZPE in kJ/mol	0.planar	-3.0			
Raw Data	0	0.2893006			
Energy in Hartree	0.planar	0.2881609			

Table S-36: Hartree-Fock single-point calculations on the planar and non-planar structures of triisopropylamine at two different geometries (MP2/aug-cc-pV(D,T)Z and B3LYP/TZVPPD) with different basis sets (aug-cc-pVXZ, X=D,T,Q,5) and extrapolations (aug-cc-pVXYZ, X=D,T,Q, Y=T,Q,5).

Triisopropylamine		HF			
raw data	in Hartree	DZ	TZ	QZ	5Z
MP2 geom	0	-407.5105397	-407.6096311	-407.6339361	-407.6395159
	planar	-407.5111084	-407.6108553	-407.6349666	-407.6405559
B3LYP geom	0	-407.5125635	-407.6109668	-407.6352143	-407.6407705
	planar	-407.512241	-407.6106051	-407.6351357	-407.6406978
MP2 geom	difference 0-p	1.493236313	3.213991559	2.705533832	2.73054677
B3LYP geom	difference 0-p	-0.84662194	-0.949736339	-0.206300054	-0.19092512
raw data	in Hartree		DTZ	TQZ	Q5Z
MP2 geom	0		-407.6246592	-407.6414984	-407.6422354
	planar		-407.6259827	-407.6424686	-407.64328
B3LYP geom	0		-407.6258905	-407.6427586	-407.6434786
	planar		-407.6255228	-407.6427681	-407.6434087
in kJ/mol					
MP2 geom	difference 0-p		3.5	2.5	2.7
B3LYP geom	difference 0-p		-1.0	0.0	-0.2

Table S-37: MP2 single-point calculations on the planar and non-planar structures of triisopropylamine at two different geometries (MP2/aug-cc-pV(D,T)Z and B3LYP/TZVPPD) with different basis sets (aug-cc-pVXZ, X=D,T,Q,5) and extrapolations (aug-cc-pVXYZ, X=D,T,Q, Y=T,Q,5).

Triisopropylamine		MP2			
raw data	in Hartree	DZ	TZ	QZ	5Z
MP2 geom	0	-409.065202	-409.4583525	-409.5763621	-409.6171845
	planar	-409.0627808	-409.4556908	-409.5741471	-409.6150101
B3LYP geom	0	-409.0662927	-409.4575651	-409.575306	-409.616044
	planar	-409.0644516	-409.4559597	-409.5735382	-409.6142983
MP2 geom	difference 0-p	-6.356696827	-6.988204644	-5.815518321	-5.708880185
B3LYP geom	difference 0-p	-4.833836934	-4.21484593	-4.641336251	-4.583159612
raw data	in Hartree		DTZ	TQZ	Q5Z
MP2 geom	0		-409.5971949	-409.6523033	-409.6568799
	planar		-409.5942554	-409.6504953	-409.6547428
B3LYP geom	0		-409.5958021	-409.6510754	-409.6556639
	planar		-409.5943066	-409.6490705	-409.6539382
in kJ/mol					
MP2 geom	difference 0-p		-7.7	-4.7	-5.6
B3LYP geom	difference 0-p		-3.9	-5.3	-4.5

Table S-38: CCSD and CCSD(T) single-point calculations on the planar and non-planar structures of triisopropylamine at two different geometries (MP2/aug-cc-pV(D,T)Z and B3LYP/TZVPPD) with different basis sets (aug-cc-pVXZ, X=D,T) and extrapolations (aug-cc-pVDTZ) and final basis set limit CCSD(T) values (MP2/aug-cc-pV(Q,5)Z+ΔCCSD(T)/aug-cc-pV(D,T)Z).

Triisopropylamine		CCSD		CCSD(T)	
raw data	in Hartree	DZ	TZ	DZ	TZ
MP2 geom	0	-409.1888128	-409.5544717	-409.2484342	-409.6362976
	planar	-409.1867996	-409.5526061	-409.2460409	-409.6339543
B3LYP geom	0	-409.1909	-409.5545469	-409.2506602	-409.6364948
	planar	-409.1892421	-409.5529259	-409.2488088	-409.6346155
MP2 geom	difference 0-p	-5.285574597	-4.898214481	-6.283784826	-6.152519228
B3LYP geom	difference 0-p	-4.352600979	-4.256023763	-4.860854914	-4.934033403
raw data	in Hartree			CCSD(T)/DZ+MP2/TQZ	CCSD(T)/DTZ+MP2/Q5Z
MP2 geom	0			-409.8355356	-409.6546538
	planar			-409.8337553	-409.652639
B3LYP geom	0			-409.8354428	-409.6533743
	planar			-409.8334277	-409.6515377
in kJ/mol					
MP2 geom	difference 0-p			-4.7	-5.3
B3LYP geom	difference 0-p			-5.3	-4.8

References

[S-1] O. V. Dolomanov, L. J. Bourhis, R. J. Gildea, J. A. K. Howard, H. Puschmann, *J. Appl. Cryst* **2009**, *42*, 339–341.

[S-2] G. M. Sheldrick, *Acta Cryst. A64* **2008**, 112–122.

[S-3] G. M. Sheldrick, *Acta Cryst. A71* **2015**, 3–8.

[S-4] a) L. Palatinus, G. Chapuis, G. *J. Appl. Crystallogr*. **2007**, *40*, 786–790; b) L. Palatinus, A. van der Lee, *J. Appl. Crystallogr*. **2008**, *41*, 975–984; c) L. Palatinus, S. J. Prathapa, S. van Smaalen, *J. Appl. Crystallogr*. **2012**, *45*, 575–580.

[S-5] G. M. Sheldrick, *Acta Cryst. C71* **2015**, 3–8.

[S-6] TURBOMOLE V7.4 **2019**, a development of the University of Karlsruhe and Forschungszentrum Karlsruhe GmbH, 1989-2007, TURBOMOLE GmbH, since **2007**; available from http://www.turbomole.com.

[S-7] a) G. Kresse, J. Hafner, *Phys. Rev. B: Condens. Matter Mater. Phys.* **1993**, *47*, 558–561. b) G. Kresse. J. Hafner, *Phys. Rev. B: Condens. Matter Mater. Phys.* **1994**, *49*, 14251–14269. c) G. Kresse, J. Furthmüller, *Phys. Rev. B: Condens. Matter Mater. Phys.* **1996**, *54*, 11169–11186. d) G. Kresse, J. Furthmüller, *Comput. Mater. Sci.* **1996**, *6*, 15–50.

[S-8] a) R. A. Kendall, T. H. Dunning, Jr., R. J. Harrison, *J. Chem. Phys.* **1992**, *96*, 6796–6806. b) D. E. Woon, T. H. Dunning, Jr., *J. Chem. Phys.* **1993**, *99*, 3730–3737. c) A. K. Wilson, D. E. Woom, K. A. Peterson, T. H. Dunning, Jr., *J. Chem. Phys.* **1999**, *110*, 7667–7676.

[S-9] A. Halkier, T. Helgaker, P. Jorgensen, W. Klopper, H. Koch, J. Olsen, A. K. Wilson, *Chem. Phys. Lett.* **1998**, *286*, 243 –252.

[S-10] J. M. L. Martin, G. De Oliveira, *J. Chem. Phys.* **1999**, *111*, 1843–1856.

[S-11] H. C. Brown, *US6248885 (B1)*, **2001**.

[S-12] K. Banert, M. Heck, A. Ihle, J. Kronawitt, T. Pester, T. Shoker, *J. Org. Chem.* **2018**, *83*, 5138−5148.

[S-13] A. McNally, B. Haffemayer, B. S. Collins, M. J. Gaunt, *Nature* **2014**, *510*, 129–133.

[S-14] A. Unsinn, M. J. Ford, P. Knochel, *Org. Lett.* **2013**, *15*, 1128–1131.

[S-15] a) S. Grimme, *J. Chem. Phys.* **2003**, *118*, 9095–9102. b) J. Antony, S. Grimme, *J. Phys. Chem. A* **2007**, *111*, 4862–4868.

[S-16] a) A. Tkatchenko, R. A. DiStasio Jr., R. Car, M. Scheffler, *Phys. Rev. Lett.* **2012**, *108*, 236402. b) A. Ambrosetti, A. M. Reilly, R. A. DiStasio Jr., A. Tkatchenko, *J. Chem. Phys.* **2014**, *140*, 18A508.

[S-17] C. Adamo, V. Barone, *J. Chem. Phys.* **1999**, *110*, 6158–6170.
